# Improving Resource Management for Unattended Observation of the Marginal Ice Zone Using Autonomous Underwater Gliders

**DOI:** 10.3389/frobt.2020.579256

**Published:** 2021-01-18

**Authors:** Zachary Duguid, Richard Camilli

**Affiliations:** ^1^Applied Ocean Physics and Engineering, Woods Hole Oceanographic Institution, Woods Hole, MA, United States; ^2^Mechanical Engineering, Massachusetts Institute of Technology, Cambridge, MA, United States

**Keywords:** autonomous underwater glider, under-ice, long-range, onboard acoustic sensing, environment state estimation, marginal ice zone, adaptive control, energy efficiency

## Abstract

We present control policies for use with a modified autonomous underwater glider that are intended to enable remote launch/recovery and long-range unattended survey of the Arctic's marginal ice zone (MIZ). This region of the Arctic is poorly characterized but critical to the dynamics of ice advance and retreat. Due to the high cost of operating support vessels in the Arctic, the proposed glider architecture minimizes external infrastructure requirements for navigation and mission updates to brief and infrequent satellite updates on the order of once per day. This is possible through intelligent power management in combination with hybrid propulsion, adaptive velocity control, and dynamic depth band selection based on real-time environmental state estimation. We examine the energy savings, range improvements, decreased communication requirements, and temporal consistency that can be attained with the proposed glider architecture and control policies based on preliminary field data, and we discuss a future MIZ survey mission concept in the Arctic. Although the sensing and control policies presented here focus on under ice missions with an unattended underwater glider, they are hardware independent and are transferable to other robotic vehicle classes, including in aerial and space domains.

## 1. Introduction

The Arctic is the most rapidly warming region on Earth and over the past several decades these rising temperatures have had a substantial impact on the region's seasonal sea-ice cover and volume (Serreze and Barry, 2011; Stammerjohn et al., 2012). Changing conditions in the Arctic have broad ramifications for the global Earth system, including rising global temperatures, as well as biological, chemical, physical, and societal impacts (Arrigo et al., 2014; Barnhart et al., 2014; Christiansen et al., 2014; Jakobsson et al., 2014; Cohen et al., 2018). Diminished sea-ice volume results in diminished latent heat thermal buffering capacity, which will further accelerate warming of the Earth's oceans (Jackson et al., 2012; Jeffries et al., 2013; Horvat and Tziperman, 2015). Thus, it is critical to accurately understand Arctic sea ice inventory. In addition to rising temperatures, positive feedback mechanisms of albedo (Curry et al., 1995) and momentum transfer (Zippel and Thomson, 2016) are causing the decline of Arctic sea-ice to accelerate. Sea-ice, and particularly the snow settling on top of sea-ice, has a characteristic albedo that is among the highest of all natural materials found on Earth's surface, causing sea-ice and snow to reflect the majority of incoming solar radiation back into the atmosphere. However, when sea-ice melts and exposes the ocean below, the low-albedo seawater absorbs the majority of the solar radiation causing the sea-surface temperature to rise, which provokes more sea-ice melting (Nicolaus et al., 2012). Additionally, as sea-ice cover declines, it is less able to dampen sea surface kinetics, amplifying the mechanical break-up of sea-ice, which leads to further melting (Zhang et al., 2015). To better understand the global sea-ice latent heat budget and the processes that govern sea-ice dynamics, it is essential to improve our understanding of Arctic sea-ice volume, the transfer of heat, and momentum at the sea-ice boundary. This requires overcoming the intrinsic challenges of sea-ice survey operations.

The Arctic Ocean remains as one of the most forbidding environments for scientific research and exploration. Historical records recount numerous expeditions during 19th and 20th centuries that met tragic ends (Hovdenak, 1935; Beattie and Geiger, 2017; Todd, 2017). Despite the advance of modern technologies, the Arctic Ocean's ice cover and weather persist as barriers, and relevant areas of sea-ice study are spatially remote, often many hundreds of kilometers away from nearest land contact or operating base, imposing significant limitations on scientific observing systems. Remote aerial and satellite observation is able to acquire surface data with the greatest spatial extent while maintaining kilometer-to-meter spatial resolution and day-to-week temporal resolution. These systems are particularly well-suited for measuring sea-ice area coverage and determining the sea-ice boundary. Satellite observation is, however, generally limited by the difficulty in accurately determining sea-ice thickness and volume (Schweiger et al., 2011), as well as the inability to assess physical characteristics such as heat and momentum transfer. Ice breakers are routinely used to observe these physical characteristics, but are costly to operate and environmentally intrusive. *In-situ* surface technologies such as moorings and ice-tethered buoys are less intrusive and able to operate unattended, but require personnel and infrastructure for on-site deployment, can be crushed between ice floes (Jackson, 2016), and only provide point measurements.

Although diesel-electric (Sverdrup and Soule, 1933) and electric (Sagalevitch, 2013) human-occupied submersibles have occasionally operated under ice cover in the Arctic since 1931, these platforms are not considered viable for under-ice research because of their limited endurance and the associated risk to the crew. Nuclear-powered submarines are perhaps the most capable of all submersible platforms for *in-situ* Arctic ice observation because of their ample underwater endurance, and in 1958 they were the first type of marine vessel to reach the North Pole (Griffin, 2013). Subsequent decades of Arctic sea-ice research have utilized nuclear submarines (Elizabeth et al., 1975; Wadhams and Horne, 1980; Wadhams, 1983; Bourke and Garrett, 1987; Rothrock et al., 1999), but an obvious downside is that these platforms are prohibitively costly to operate and are the exclusive domain of only a few of the world's militaries. Remotely operated vehicles (ROVs), which are now a far more accessible technology for the scientific community, have demonstrated the utility for detailed ice pack observation at appropriate standoff distances from an ice breaker's path (German et al., 2014), and along basin-scale science transects (Nicolaus and Katlein, 2013), but they require the support of surface vessels or ice camps for operation and their horizontal speed and range are limited by the vehicle tether, making scaling up for wide area coverage with high spatial and temporal resolution challenging. In the nearly 50 years since the first autonomous underwater vehicle (AUV) was operated under ice (Francois and Nodland, 1972), the usage of these vehicle platforms has progressively increased (Thomas, 1986; Thorleifson et al., 1997; Kunz et al., 2009; McPhail et al., 2009; Kaminski et al., 2010; Kim et al., 2013; Williams et al., 2015; Kimura et al., 2016; Kukulya et al., 2016; Graham et al., 2019; Spain et al., 2019). With notable exceptions (Thorleifson et al., 1997; McPhail et al., 2009; Kaminski et al., 2010; Furlong et al., 2012; Kimura et al., 2016), most AUVs do not possess the required endurance to conduct long-range Arctic missions. Furthermore, under-ice navigation amplifies the drawbacks of range limitation because in this GPS-denied environment, the vehicle is unable to surface at will for a position fix and if valid acoustic beacon fixes are not available, accumulating navigation error can cause the vehicle to become “increasingly lost” (Plueddemann et al., 2012), decreasing the likelihood that an appropriate contingency plan can be successfully executed within the limitations of the vehicle's dwindling power budget and potentially critical temporal constraints. Although AUVs can overcome range limitations by scaling up in size, unit costs of robotic underwater vehicles tend to correlate with displacement, and operating costs are influenced substantially by launch and recovery infrastructure requirements, leading to an increasing trend in capital and operating costs.

**Figure 1:**
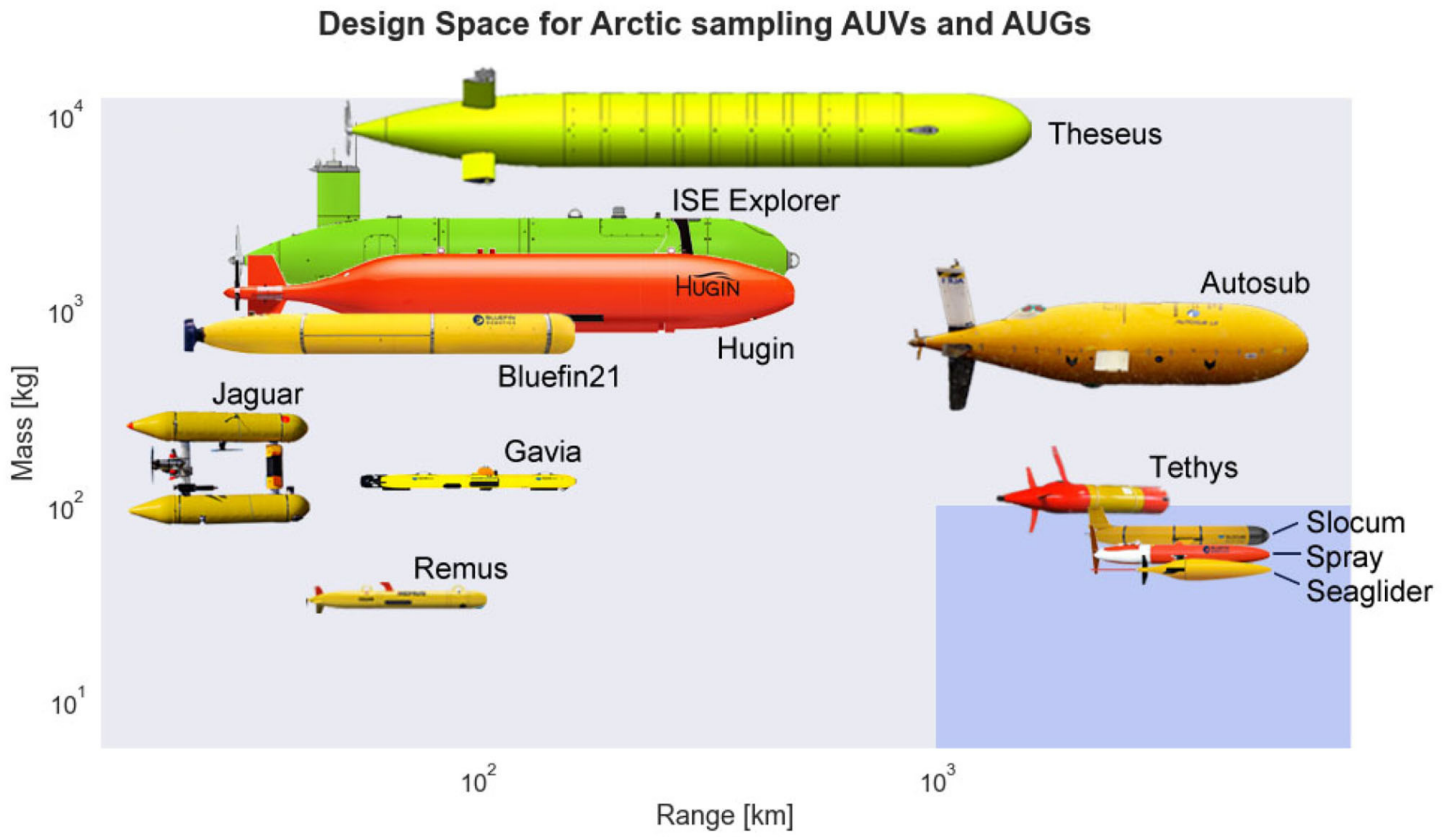
Overview of design space for under-ice surveying autonomous underwater vehicles (AUVs) and gliders in terms of mass and range. This trade space includes several size classes of underwater vehicles: extra large vehicles such as Theseus (Butler and den Hertog, 1993), large vehicles such as ISE Explorer, Hugin, Bluefin21, and Autosub (Kaminski et al., 2010; Furlong et al., 2012; Lehmenhecker and Wulff, 2013; Graham et al., 2019), medium vehicles such as Jaguar, Gavia, and Tethys (Wadhams and Doble, 2008; Kunz et al., 2009; Bellingham et al., 2010), and small vehicles such as Remus, Slocum, Spray, Seaglider (Sherman et al., 2001; Kukulya et al., 2010; Jones et al., 2014; Lee et al., 2017). The blue highlighted region indicates the area of interest within the design space: vehicles of modest size that can support meaningful payloads while maintaining long-range capabilities. The top-right corner of each vehicle image indicates its approximate numerical value within the design space. The scaling and relative position of the vehicle images is approximate.

Despite being relatively slow and minimally navigated, gliders offers a promising compromise between range, cost, and observational capability. Their inherent capability for high-endurance and unattended operation without need for acoustic navigation beacons has enabled this class of vehicle to successfully complete multiple trans-Atlantic missions (Willis, 2009; Ramos et al., 2018) and routinely operate in polar waters (Miles et al., 2016; Lee et al., 2017; Zhou et al., 2019). With appropriate modification, these vehicles may be viable for long-range Arctic missions requiring round-trip transits from ports of convenience. Scaling up to persistent synoptic observation using unattended underwater platforms will require miserly power budgets coupled with improved navigation, low unit costs, and onboard active sensing. Figure 1 illustrates the performance envelope of autonomous vehicle designs for under-ice operation in terms of maximum range and vehicle displacement, while highlighting the target region that would enable long-range unattended Arctic missions without need for costly launch and recovery infrastructure.

These infrastructure constraints effectively limit vehicle size and onboard energy storage, which directly influence range and science capabilities. Despite these constraints, we propose that energy-optimized vehicle behaviors can be applied to a commercially available hybrid glider that enables efficient transit and unattended operation with range and endurance appropriate for extended observation of sea-ice, without the need for acoustic beacons. Although the methods and policies proposed here are extensible to many classes of autonomous underwater vehicles and mission scenarios, we focus specifically on unattended subsea observation of the marginal ice zone (MIZ) during seasonal advance and retreat because its associated dynamics (e.g., momentum flux, temperature and salinity gradients, and ice characteristics) are difficult to observe with conventional technologies. In this scenario, we envision a vehicle that can be launched from a coastal port of opportunity and be capable of unattended round trip transit through ice-free areas (order 1,000 km) to observe MIZ regions of interest for repeated short under-ice excursions (order 10 km) that include water column profiling extending down to 1 km during a campaign lasting weeks to months. This mission scenario does not contemplate launch or recovery from within ice covered regions, but instead utilizes established acoustic Doppler localization techniques to enable the glider to periodically transit back out to a previously known open water region. The absence of ice can be confirmed using the vehicles embedded imaging sonar interpretation process prior to attempted surfacing for opportunistic position fixes, mission updates, and low-bandwidth transfer of MIZ observation data.

## 2. Materials and Methods

### 2.1. Review of Hardware Components

**Figure 2:**
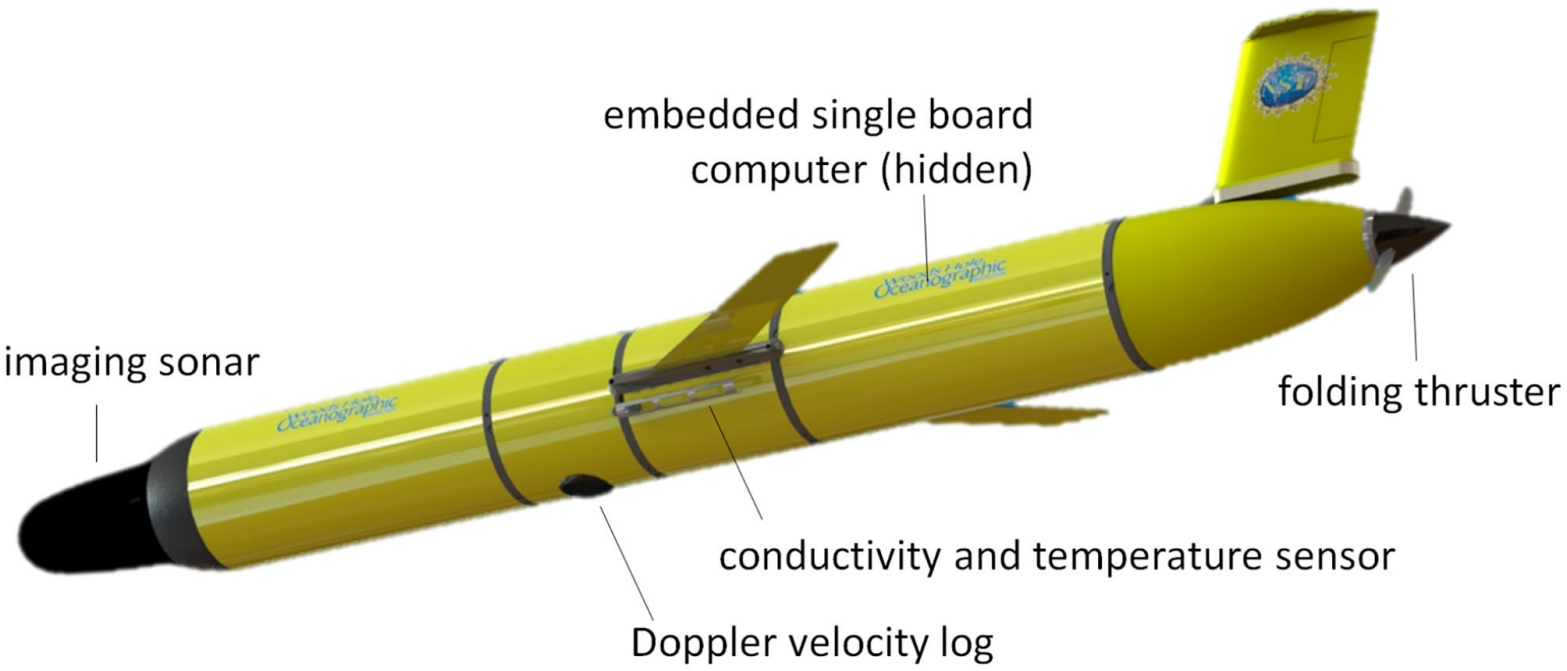
CAD rendering of the *Polar Sentinel* glider, showing relative locations of the mechanically scanned imaging sonar, Doppler velocity log, conductivity and temperature sensor, embedded single board computer, and folding thruster.

Our focus on active management of onboard resources centers principally on energy efficiency for improving the overall performance of *Polar Sentinel* (Figure 2), a hybrid glider with 10 W folding thruster [Slocum G3 electric, Teledyne Webb Research], equipped with a 600 kHz phased array Doppler velocity log (DVL) [Pathfinder, Teledyne RDI], 700 kHz Mechanically Scanning Imaging Sonar (MSIS) [Micron, Tritech] housed within a modified nosecone, a payload Conductivity, Temperature, and Depth (CTD) sensor [GPCTD, Sea-Bird Scientific], and an environmental state estimator and continuous replanner operating on an embedded single board computer [Pi-Zero, Raspberry Pi], referred to as the Backseat Driver (BSD) computer. This architecture utilizes real-time environmental state information derived from its commercially available low-power acoustic sensors. To further improve energy efficiency, we consider low-level optimization behaviors for hybrid propulsion and acoustic sensing in conjunction with onboard data interpretation that allows for efficient information transfer during vehicle surfacing.

Glider power expenditure can be divided into two primary categories: propulsive and hotel. As a hybrid vehicle, propulsive power includes power draw for the buoyancy engine and power draw to drive the propeller. Hotel electric power, or hotel load, consists of all non-propulsive systems running onboard the glider, including the flight computer, active control systems, acoustic sensors, and communication equipment. In this section, we review the hardware components contained in our proposed architecture, introduce the problem of energy optimization in the context of vehicle transit and survey operations, describe an algorithm for real-time environmental state estimation, and present energy management policies that can be embedded within the glider for continuous optimization.

Glider hardware components can be divided into four main categories: vehicle propulsion and control systems, sensors, computers, and communication. A summary of the glider's hardware components and corresponding power requirements are given in Table 1.

**Table 1:** Review of hardware components including instantaneous power draw when the component is active, the expected duty cycle during a typical glider mission, and the average power draw over the course of the mission.

**Hardware component**	**Inst. power**	**Duty cycle**	**Avg. power**
	**[W]**	**[%]**	**[W]**
Thruster (min)	1.0	98.4	0.98
Thruster (max)	10.0	98.4	9.84
Buoyancy engine	101.0	1.6	1.60
Pitch controller	2.2	0.9	0.02
Rudder	2.4	1.0	0.02
Altimeter	0.4	41.2	0.17
DVL	2.0	100.0	2.00
MSIS	3.0	100.0	3.00
CTD	0.1	100.0	0.14
Micro IMU	0.4	100.0	0.40
Flight computer	0.2	100.0	0.16
BSD	0.4	100.0	0.40
Radio modem	21.0	0.0	0.00
Satellite comms	6.0	1.0	0.06

*Hardware components fall into four main categories: vehicle propulsion and control systems, sensors, computers, and communication*.

The hotel load for the glider refers to all non-propulsive power systems. For normal flight conditions, not all of the components mentioned in Table 1 are necessary. For example, the communication components are only used when the glider is at the surface, which is a small fraction of total mission time. By toggling different components on and off, we can define a set of possible operating conditions. Two operating modes that are commonly defined for underwater vehicles conducting science missions are *transit mode* and *survey mode*. When the glider is in transmit mode, scientific and non-essential systems are turned off to limit power expenditure while traveling to science targets. Conversely, when the glider is in survey mode, analytical sensors are turned on to enable observation of the science target. Hotel configuration can, however, be expressed as gradations between transit and survey mode, instead of just binary states. For example, in instances of moderate ocean currents, it may be beneficial to operate the DVL while transiting to improve glider navigation via active steering to counteract cross-track ocean currents even though the hotel load is increased. Further treatment of hotel load optimization is discussed in section 2.8. We define transit mode and survey mode as the upper and lower bounds of the glider's hotel load, without including the power draw from communication equipment. For the *Polar Sentinel* glider, this yields an average power demand for transit and survey mode of 0.37 W and 6.37 W, respectively.

Slocum gliders can be equipped with commercially available *extended endurance* pack configurations composed of lithium primary cells, providing approximately 10 kW h at ambient Arctic ocean temperatures. Although lithium primary batteries are not rechargeable, they allow for a threefold increase in the glider's onboard energy capacity compared to lithium ion secondary batteries to help meet the range requirements of an Arctic mission. Both classes of lithium battery chemistry have thermal characteristics that are superior to most other battery chemistries, making them better suited for Arctic missions where water temperature is approximately −2 °C.

### 2.2. Objective Function: Minimize Transport Cost

Several objectives must be considered simultaneously when planning glider missions. For example, objectives pertaining to range, endurance, localization, science, safety, communication, and rendezvous must be considered. Each of these objectives must be satisfied via the utilization of one common resource: the energy carried onboard the glider. Therefore, efficient allocation of energy is universally beneficial for extending glider missions.

When developing a glider mission plan, the notion of resource management can be defined as one of several objectives that is considered in the context of a multi-objective optimization problem. This can be expressed as:

$$\begin{array}{l} \underset{\text{u}}{\min}\left[f_{1}\left(\text{u}\right),f_{2}\left(\text{u}\right),\ldots ,f_{k}\left(\text{u}\right)\right]\\ \text{s.t. }\text{u}\in U \end{array}$$

where *k* is an integer number of objective functions, *f*_*i*_ is the *i*th objective function, **u** is a decision vector, and *U* is the feasible set of decision vectors. Although constraints such as vehicle dynamics limit the feasible set of decision vectors, multi-objective optimization is challenging and often intractable due to competing objectives and uncertainty in environmental variables such as ocean current conditions and sea-ice cover. For context, let us consider that the glider must decide whether or not an additional sensor, such as an imaging sonar, should be turned on. The resource management objective would be minimized if the sensor remains off, but the science objective would be minimized if the sensor is turned on. Although glider propulsive commands can be optimized with respect to scientific objectives alone (Smith et al., 2011), optimization with respect to multiple non-commensurate objectives is challenging (Schwartz et al., 2002). Additionally, since environmental states are generally uncertain, it is difficult to provide guaranteed performance for a particular mission plan. Methods designed to account for the relative importance of competing objectives during the mission include the valuated state space (Jenkins et al., 2003) method and interval programming method (Benjamin et al., 2010).

Rather than considering resource management in the context of global optimization over glider control variables, we consider resource management as applied to a decoupled version of the problem where a subset of the control variables are provided *a priori* by some high-level mission planner and hotel load optimization policy. Let us assume that the high-level mission planner (details of which are outside the scope of this paper) is responsible for general route parameters such as sequences of science goal points. The hotel load optimization procedure is responsible for duty cycling the glider hardware components in response to vehicle and environmental state. Then, given the high-level mission plan and hotel load, the propulsive system of the glider can be optimized with respect to energy conservation. We refer to this low level propulsive optimization as *adaptive velocity control* (AVC), which seeks to minimize the transport cost of the glider. We define transport cost in terms of Joules expended per meter traveled, which unlike transport economy (Jenkins et al., 2003) is independent of vehicle weight in order to avoid biases favoring increased vehicle displacements without consideration of supporting infrastructure requirements, as well as associated capital and operating costs.

By decoupling the glider control problem into hierarchical steps, the propulsive system can be optimized for transport cost independently of other glider control choices. The decision vector **u** can then be divided into two subcomponents: $\text{u}={\left[{\text{u}}_{\text{p}},{\text{u}}_{\text{h}}\right]}^{T}$, where **u**_**p**_ refers to control variables of the glider's propulsive system and **u**_**h**_ refers to the control variables of the glider's hotel system. Then, AVC can be written as:

$$\begin{array}{l} {\text{u}}^{*}=\underset{\text{u}}{arg min}\left[f_{TC}\left(\text{u}\right)\;|\;{\text{u}}_{\text{h}}\right]\\ \text{s.t. }\text{u}\in U \end{array}$$

where *f*_*TC*_ represents the transport cost of the glider and is independent of time. Assuming that the vertical component of ocean currents is negligible compared to the horizontal component, we define the transport cost objective function *f*_*TC*_ to be equal to the total energy expended per horizontal distance traveled, which can then be written as the total power draw divided by horizontal over-ground velocity:

(1) $$\begin{array}{l} f_{TC}=\frac{\Delta E}{\Delta x}=\frac{\text{Total Power}}{\text{Horizontal Velocity}} \end{array}$$

Minimizing *f*_*TC*_ is the same as maximizing $\frac{1}{f_{TC}}=\frac{\Delta x}{\Delta E}$, which is equivalent to maximizing the total range of the glider given a finite energy supply. It is important to reiterate that Δ*E* refers to the total energy expended by the glider, not just the energy expended via the glider propulsive system. As a result, the science payload influences optimal vehicle velocity (Bradley, 1992; Jenkins et al., 2003; Hobson et al., 2012).

### 2.3. Hybrid Propulsion

To optimize transport cost, we model the glider's propulsive system, which is able to utilize both the buoyancy engine and propeller thruster for propulsion. This architecture reaps the benefits of both propulsive strategies: efficiency at low speeds via the buoyancy engine and freedom of speed variability via the propeller thruster. The hybrid thruster is particularly useful because it enables the glider to operate (i.e., make headway) despite adverse or cross-track currents, whereas a glider equipped with buoyancy engine alone would be susceptible to being swept off-course. Prior work by Jenkins et al. suggests a strategy wherein gliders utilize high-speed transport to mitigate the negative impact of adverse or cross-track currents (Jenkins et al., 2003). The ability to travel at higher speeds is especially useful in the context of sea-ice surveys because higher speed may allow the glider to evade dynamic sea-ice cover in order to reach a safe surfacing location, which is particularly important for operation during seasonal ice advance.

In addition to the wider velocity envelope, hybrid thrust improves flexibility for glider path planning by not requiring that the glider rely entirely on the sawtooth path associated with the buoyancy engine. The glider can instead travel at an arbitrary depth band without loss of speed or efficiency, which may be beneficial for scientific observation, navigation and localization, obstacle avoidance, or improved efficiency in shallow waters. Using the buoyancy engine in conjunction with the thruster avoids a drawback common to AUVs of expending energy to achieve neutral stability. AUVs often must contend with this because they are ballasted slightly positively buoyant as a safety precaution (Bellingham et al., 1995; Jenkins et al., 2003). Since the glider does not have access to elevator control, it can be challenging to stabilize with thruster and pitch controller alone (Claus et al., 2012). By providing a stabilizing force via buoyant loading of the glide surfaces, the buoyancy engine improves the efficiency of the thruster. Finally, this flexibility of two parallel propulsive strategies makes the glider more redundant and tolerant to propulsion system failure during the mission, increasing the probability of vehicle survivability.

To express the transport cost of the hybrid glider, the power and velocity of both the buoyancy engine and the propeller thruster must be modeled. By modeling both modes of glider propulsion, we attain an expression for the glider's through-water velocity **V**_*tw*_:

(2) $$\begin{array}{l} {\text{V}}_{tw}={\text{V}}_{buoy}+{\text{V}}_{thr} \end{array}$$

where **V**_*buoy*_ represents the through-water velocity from the buoyancy engine and **V**_*thr*_ represents the through-water velocity from the thruster. However, to obtain an expression for the glider's over-ground velocity **V**_*og*_, the effect of ocean current velocity **V**_*oc*_ must be included:

(3) $$\begin{array}{l} {\text{V}}_{og}={\text{V}}_{tw}+{\text{V}}_{oc} \end{array}$$

Here, **V**_*og*_ and **V**_*oc*_ are vectors in the absolute world frame, while **V**_*tw*_ is a vector in the relative vehicle frame. Each vector can be broken into Eastward, Northward, and vertical components:

(4) $$\begin{array}{l} {\text{V}}_{og}={\left(\begin{array}{ccc} u_{og} & \nu_{og} & w_{og} \end{array}\right)}^{T} \end{array}$$

(5) $$\begin{array}{l} {\text{V}}_{tw}={\left(\begin{array}{ccc} u_{tw} & \nu_{tw} & w_{tw} \end{array}\right)}^{T} \end{array}$$

(6) $$\begin{array}{l} {\text{V}}_{oc}={\left(\begin{array}{ccc} u_{oc} & \nu_{oc} & w_{oc} \end{array}\right)}^{T} \end{array}$$

Since minimizing the transport cost of the glider is dependent on the over-ground velocity of the vehicle, the glider must be capable of estimating ocean current velocities in real time. This ocean current sensing process is discussed in detail in section 2.6. For now, we assume that ocean current velocity is a known variable.

Similar to the modeling of the through-water velocity of the glider, the glider propulsive power *P*_**p**_ can be expressed as the sum of the buoyancy engine power draw *P*_*buoy*_ and the thruster power draw *P*_*thr*_. Then, the total power draw *P*_*tot*_ can be expressed as the sum of the propulsive power and hotel power *P*_**h**_:

(7) $$\begin{array}{l} P_{tot}=P_{\text{p}}+P_{\text{h}} \end{array}$$

(8) $$\begin{array}{l} =P_{buoy}+P_{thr}+P_{\text{h}} \end{array}$$

#### 2.3.1. Buoyancy Engine Model

The buoyancy engine allows the glider to travel through the water column while generating forward motion in a sawtooth trajectory, with the glider inflecting downward once it reaches the top of its flight band and inflecting upward upon reaching the bottom of its flight band. Through the use of ambient hydrostatic pressure, the glider only needs to actively pump ballast at the lower inflection points, enabling low energy expenditure during the majority of the trajectory. Glider designs such as the Slocum and Seagliders utilize isopycnal hulls with compressibility characteristics roughly matching seawater in order to minimize buoyancy engine energy expenditure (Webb, 2006). The buoyancy engine's efficiency decreases, however, in proportion to a narrowing flight band because a narrowing flight band corresponds with an increase in the frequency of glider inflections. Thus, efficiency is highest when the flight band includes the full depth range of the glider. Equation (9) expresses the average power draw from the buoyancy engine, describing the amount of energy expended Δ*E* per a given amount of time Δ*t*, where *E*_*pump*_ is the energy cost for operating the ballast pump during bottom inflections, Δ*z* is the size of the depth band in which the glider is operating, and *w*_*og*_ is the vertical component of the glider's over-ground velocity. In general, *E*_*pump*_ can be modeled as a function of depth *z*. For now, we assume that the glider's depth-band is predetermined, but a routine for optimizing depth-band is discussed in section 2.5.

(9) $$\begin{array}{l} P_{buoy}=\frac{\Delta E}{\Delta t}=\frac{E_{pump}}{\left[\frac{2\left(\Delta z\right)}{\bar{w_{og}}}\right]} \end{array}$$

Analytical models adapted from Jenkins et al. (2003) and Scholz et al. (2005) indicate that the through-water velocity contribution from the buoyancy engine can be expressed as a polynomial function of pitch angle ϕ, as shown in Equation (10). Detailed studies of Slocum Glider dynamics by Eichhorn et al. (2020) indicate that transport cost is minimized at pitch angles of roughly 15° when glider is driven by the buoyancy engine alone, although steeper angles yield higher horizontal speeds.

(10) $$\begin{array}{l} ∥{\text{V}}_{buoy}∥=\left(1.13\times 10^{-1}\right)+\left(1.55\times 10^{-2}\right)\cdot \phi -\left(2.17\times 10^{-4}\right)\cdot \phi^{2} \end{array}$$

#### 2.3.2. Propeller Thruster Model

While the buoyancy engine is efficient for travel at relatively low speeds with large depth bands, the thruster is better suited for missions requiring higher speeds or narrow flight bands. An expanded vehicle dynamics envelope using a combination of buoyancy and thruster driven propulsion provides more flexibility for mission planning and adaptive control to contend with adverse environmental conditions often encountered in the Arctic. Large portions of the Arctic basin are on continental shelves and have shallow depths. For example, much of the Chukchi Sea is between 25 m and 50 m deep. When including an appropriate safety margin for possible sea-ice cover and inflection at depth, the resulting flight band is narrow and energetically expensive for buoyancy engine transit. Although gliders are generally designed to travel throughout their entire depth range during transit, there are control methods that can be used for hybrid gliders to travel at constant depths. Claus and Bachmeyer demonstrate the ability to use a linear reduced order model of glider dynamics paired with a linear quadratic regulator controller to minimize energy loss due to lift-induced drag from excess buoyant force (Claus and Bachmayer, 2016).

The thruster power draw *P*_*thr*_ can be modeled as a function of commanded thruster speed ∥**V**_*thr*_∥ and can be divided into two components: the power draw for operating the propeller itself *P*_*propeller*_, and the power draw for operating the associated motor controller *P*_*controller*_. Equation (11), which is used by the glider's embedded vehicle controller, accounts for the propeller and motor power dissipation. Equation (12) is a linear approximation of the motor controller's dissipation and other losses, including wire-line resistance, inductive, and frictional (Texas Instruments, 2012; Claus, 2014). Taken in combination, these equations express the total power draw of the thruster, as shown in Equation (13).

(11) $$\begin{array}{l} P_{propeller}={\left(\frac{∥{\text{V}}_{thr}∥}{0.45}\right)}^{2.56} \end{array}$$

(12) $$\begin{array}{l} P_{controller}=0.10\cdot P_{propeller}+0.32 \end{array}$$

(13) $$\begin{array}{l} P_{thr}=P_{propeller}+P_{controller} \end{array}$$

**Figure 3:**
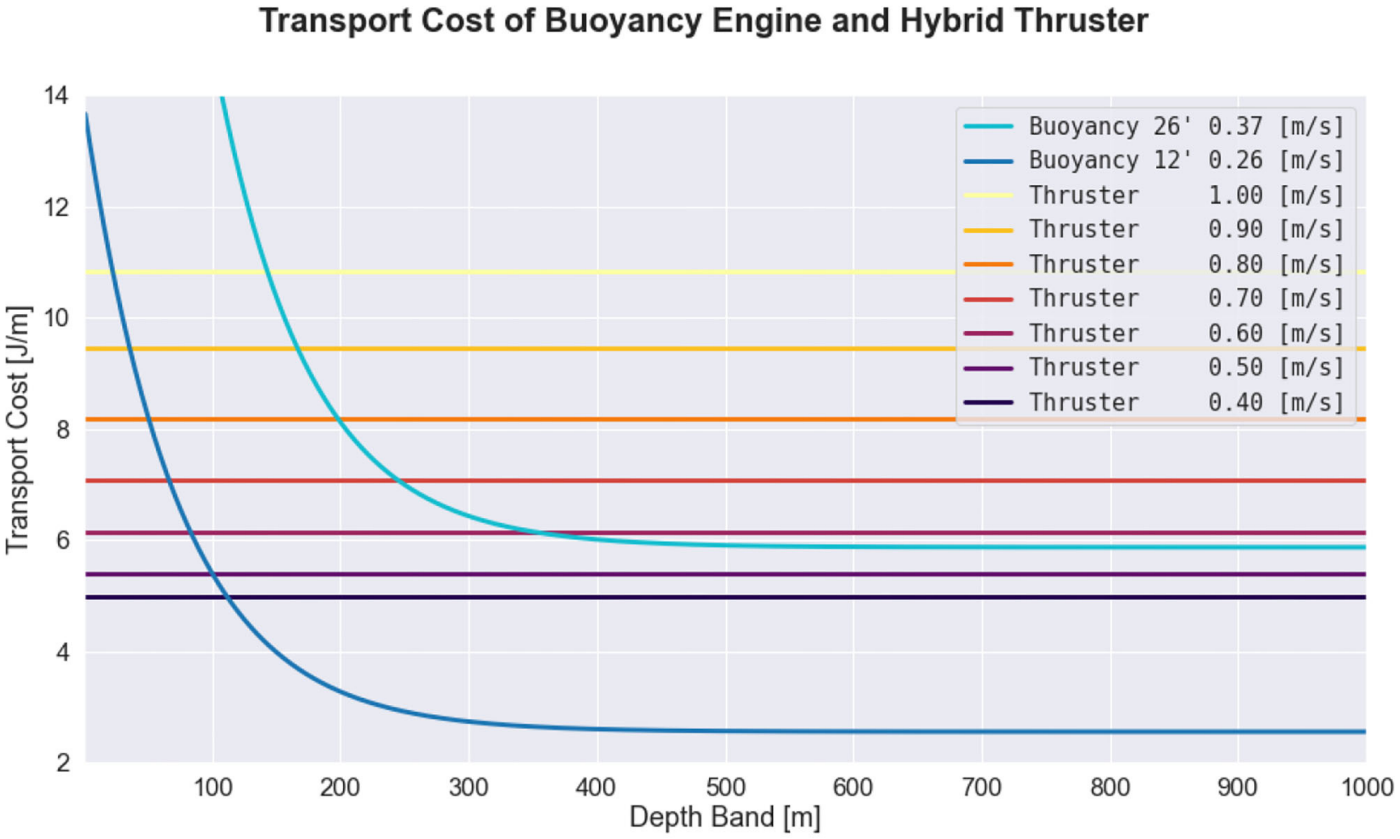
Comparison of transport cost between the buoyancy engine and the thruster when the glider is operating in transit mode, where transport cost is defined as energy expended per horizontal distance traveled. The blue curves correspond to the transport costs of the buoyancy engine at 12° and 26° pitch angles, while the yellow, orange, and red curves correspond to the thruster, which is assumed to be traveling at level flight. The average power demand of the buoyancy engine depends on ballast pumped (which is conventionally held constant) and duty cycle (which is dependent on pitch angle and depth band), while the average power demand of the thruster depends only on the thruster speed.

Figure 3 shows the comparison between buoyancy engine and thruster efficiency, where efficiency is given by the transport cost of each propulsive mechanism in isolation. Note that the two propulsive modes have different dependencies for efficiency, in particular, the thruster is most efficient at approximately 0.4 m s^-1^, independent of depth band. In contrast, the efficiency of the buoyancy engine is dependent on depth band, and tends toward greater efficiency at pitch angles that are relatively shallow in comparison to conventional missions using 26° pitch, which is consistent with polar curves estimated in the literature (Jenkins et al., 2003; Eichhorn et al., 2020). This figure suggests that a fairly broad thruster velocity envelope is more efficient than the buoyancy engine when operating within a narrow depth band. However, it is important to note that the efficiency model for the thruster does not include the energy cost associated with the instability that arises from maintaining constant depth, nor does the buoyancy model account for inefficiencies attributable to accelerations associated with vehicle pitch inflection (Deutsch et al., 2020). Although these curves suggest that propeller-generated thrust provides an advantage over buoyancy engines in regions with narrow depth band, unlike the conventional approaches that attempt to minimize lift-induced drag by minimizing net positive buoyancy (as is common with AUVs), these two modes of thrust can be used in tandem such that the buoyancy engine provides both propulsive force and stability via wing loading to assist the thruster. Additionally, these efficiency curves do not take into account the effect of science payload or ocean currents. For increase science payload or adverse ocean currents, transport cost may be minimized at higher speeds.

### 2.4. Adaptive Velocity Control

Using these models of the glider's propulsive components, adaptive velocity control (AVC) can be considered, where AVC solves the transport cost minimization problem stated in section 2.2. In doing so, AVC is first applied to a one dimensional (1D) model, and then a three-dimensional (3D) model, of glider dynamics.

In the 1D model, the glider travels in a straight line and the ocean current is either entirely favorable or entirely adverse, meaning there is no cross-track ocean current. Since the glider travels in a straight line at constant depth, the buoyancy engine is not considered in the 1D model. The transport cost for the 1D dynamics model is given in the following equation :

(14) $$\begin{array}{l} f_{TC}=\frac{P_{tot}}{u_{og}}=\frac{P_{\text{p}}+P_{\text{h}}}{u_{tw}+u_{oc}}=\frac{P_{thr}+P_{\text{h}}}{u_{thr}+u_{oc}} \end{array}$$

Here, the hotel load *P*_**h**_ and ocean currents *u*_*oc*_ are assumed to be given, and the glider propulsive control vector considers only the commanded velocity of the thruster, **u**_**p**_ = (*u*_*thr*_). Thus, the optimal through-water velocity of the glider is given by argument minimum of the transport cost objective function:

(15) $$\begin{array}{l} {\text{u}}^{*}=\underset{u_{thr}}{arg min}\left[\frac{P_{thr}+P_{\text{h}}}{u_{thr}+u_{oc}}\right] \end{array}$$

**Figure 4:**
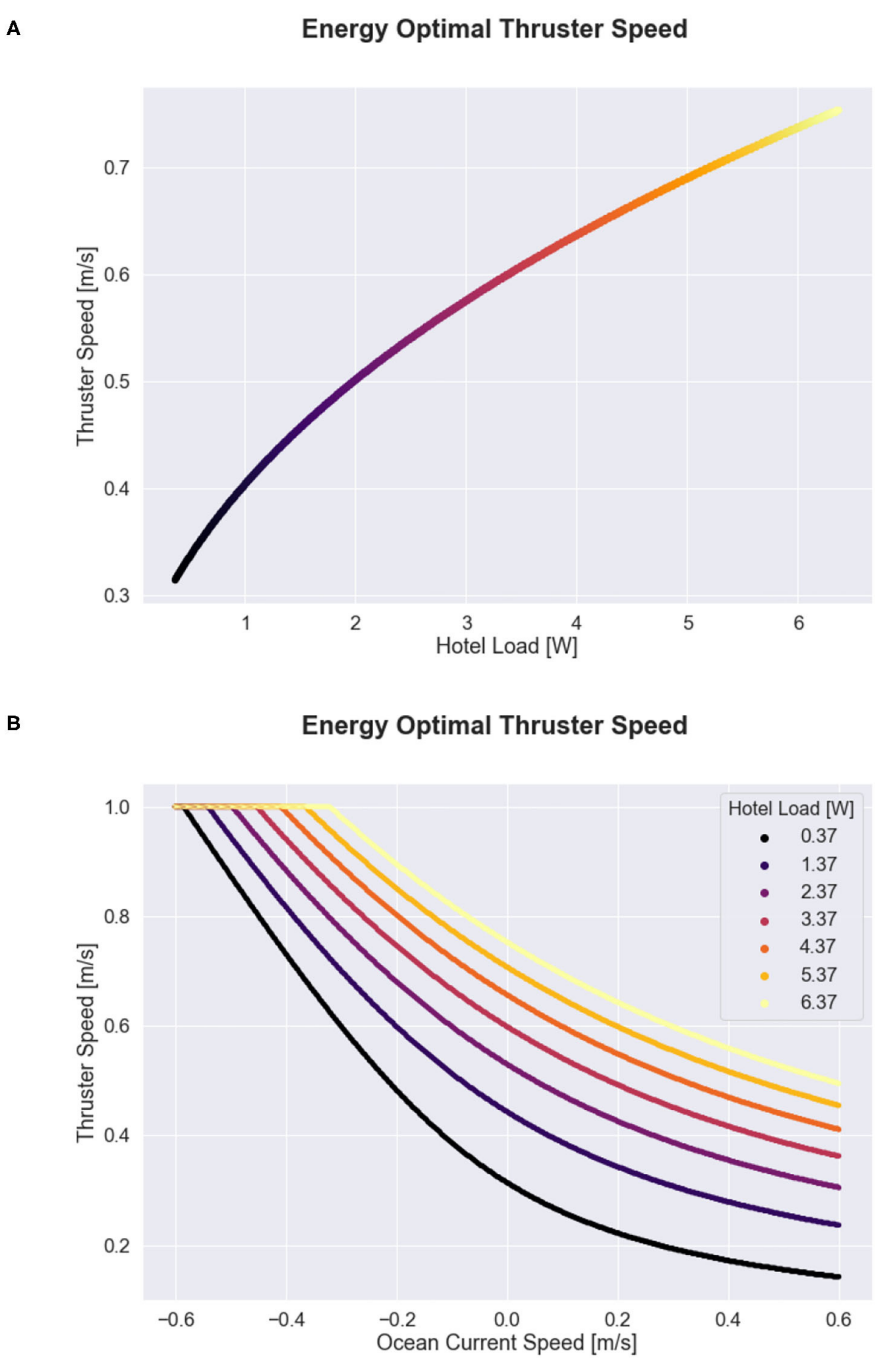
Energy optimal propulsive speed as a function of hotel load and ocean current conditions for the 1D case. **(A)** Shows optimal speed as a function of hotel load alone. **(B)** Shows the optimal speed as a function of ocean current velocity for a range of possible hotel scenarios. Color in both subplots indicates hotel load, where hotel load values vary between transit mode (0.37 W) and survey mode (6.37 W).

The optimal thruster velocity for varying hotel loads and ocean current conditions is shown in Figure 4. The figure shows that the optimal glider speed increases with hotel load and decreases with favorable ocean current currents. When there is a strong adverse current, the optimal speed for all hotel load scenarios approach the glider's maximum speed of 1.0 m s^-1^, which requires approximately 10 W of power to support.

To represent glider transport cost in a 3D domain, however, buoyancy engine dynamics and 3D ocean currents must be considered. For clarity, the process of minimizing transport cost in the 3D domain does not yield an energy-optimal route plan. Instead, it is assumed that a route plan as already been established, and then the AVC process optimizes glider propulsive parameters to minimize energy expenditure per horizontal distance traveled along the route. This optimization considers the impact of hotel load and 3D ocean currents. Additionally, it is assumed that glider pitch ϕ is predetermined based on estimated efficiency and vehicle dynamic constraints, as described by Jenkins et al. (2003), Cooney (2011), Eichhorn et al. (2020), and Deutsch et al. (2020). For now, depth band is assumed to be predetermined as well, based on the seafloor bathymetry, but the energy-optimal selection of glider depth band is revisited in section 2.5.

To express AVC in the 3D domain, some additional variables are required. First, let ψ be the glider heading, η be the ocean current heading, and δ = η − ψ be the relative alignment of the ocean currents with respect to the glider heading. Then, ∥**V**_*tw,h*_∥ represents the glider through-water speed in the horizontal plane, ∥**V**_*oc,h*_∥ represents the ocean-current speed in the horizontal plane, and ∥**V**_*og*,ψ_∥ represents the glider over-ground speed in the desired heading ψ direction, as shown in Equations (16), (17), and (18), respectively.

(16) $$\begin{array}{l} ∥{\text{V}}_{tw,h}∥={\left(u_{tw}^{2}+\nu_{tw}^{2}\right)}^{\frac{1}{2}} \end{array}$$

(17) $$\begin{array}{l} ∥{\text{V}}_{oc,h}∥={\left(u_{oc}^{2}+\nu_{oc}^{2}\right)}^{\frac{1}{2}} \end{array}$$

(18) $$\begin{array}{ll} ∥{\text{V}}_{og,\psi }∥ & =(∥{\text{V}}_{tw,h}{∥}^{2}-{\left[∥{\text{V}}_{oc,h}∥\cdot \sin\delta \right]}^{2}{)}^{\frac{1}{2}}+∥{\text{V}}_{oc,h}∥\cdot \cos\delta \end{array}$$

Equation (18) encodes the impact of cross-track currents, ∥**V**_*oc,h*_∥·sinδ, and long-track currents, ∥**V**_*oc,h*_∥·cosδ, on the glider over-ground speed in the desired heading direction. Note that the vertical component of ocean current velocity does not affect speed over-ground because it is perpendicular to the horizontal ground plane. However, the vertical component of ocean current affects the power consumption of the buoyancy engine, as shown in Equation (9).

Leveraging expressions for buoyancy engine power draw, thruster power draw, and glider over-ground speed, from Equations (9), (13), and (18), respectively, transport cost can be written for the 3D glider dynamics model:

(19) $$\begin{array}{l} f_{TC}=\frac{P_{tot}}{∥{\text{V}}_{og,\psi }∥}=\frac{P_{\text{p}}+P_{\text{h}}}{∥{\text{V}}_{og,\psi }∥}=\frac{P_{buoy}+P_{thr}+P_{\text{h}}}{∥{\text{V}}_{og,\psi }∥} \end{array}$$

If pitch and depth band are given, the buoyancy engine dynamics are fully defined, so thruster velocity remains as the sole decision variable that AVC optimizes over, **u**_**p**_ = (∥**V**_*thr*_∥). AVC can then be written as the argument minimum over the 3D transport cost equation:

(20) $$u_{p}{\;}^{*}=\underset{{\;}_{\left\|V_{thr}\right\|}}{arg min}\left[\frac{P_{buoy}+P_{thr}+P_{h}}{\left\|V_{og,\psi }\right\|}\right]$$

**Figure 5:**
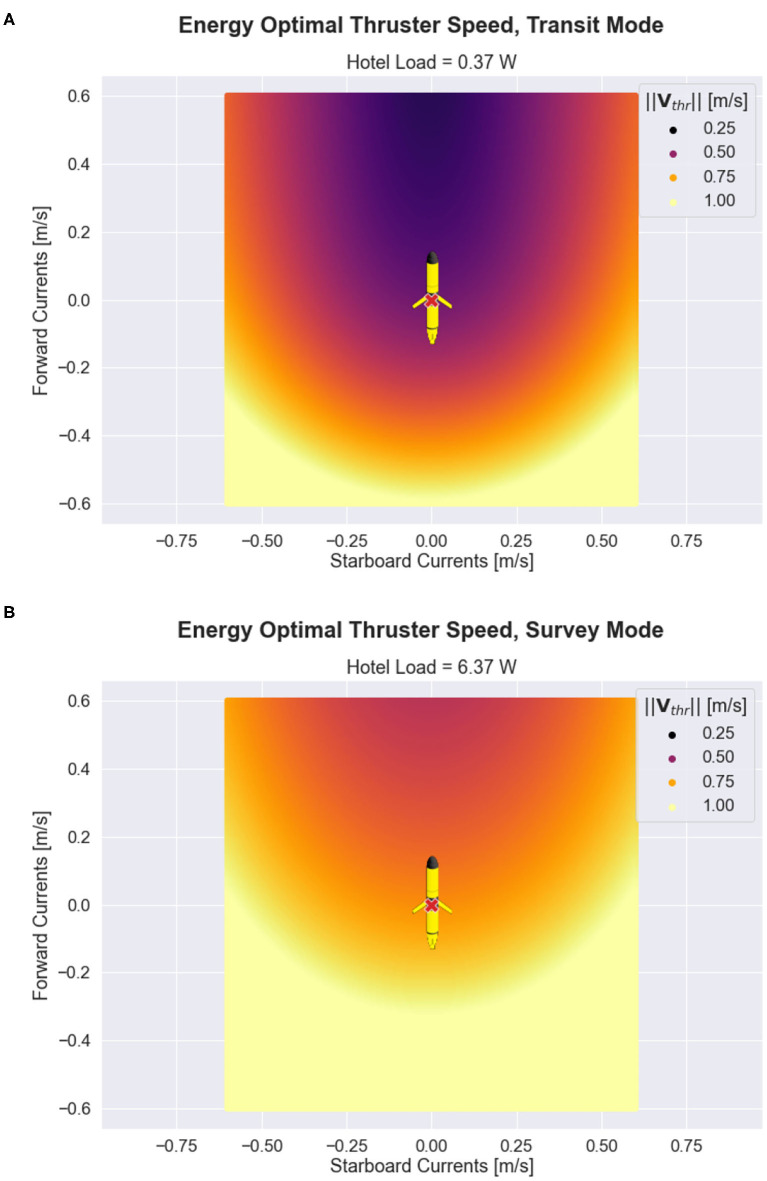
Adaptive velocity control (AVC) in 2D for ocean current speeds ≤ 0.6ms−1. In each subplot, the x-coordinate represents the starboard (cross-track) component of the ocean current velocity, and the y-coordinate represents the forward (long-track) component of the ocean current velocity. The glider is centered at the origin. The color shown in **(A,B)** represent the optimal glider thruster water speed for transit and survey hotel, according to the AVC method presented in Equation (20).

Figures 5A,B echo the results obtained in the 1D case, namely that it is energetically beneficial for the glider to speed up in the presence of adverse currents or increased hotel loads and to slow down in the presence of favorable currents or decreased hotel loads. Additionally, the figure conveys the impact of cross-track ocean currents: the glider must increase its velocity significantly to counteract cross-track currents while still maintaining forward progress.

Since the optimal through-water velocity exactly counteracts cross-track currents while maintaining forward progress, AVC inherently includes active steering to minimize cross-track and long-track drift caused by ocean currents. Thus, the active steering aspect of AVC leads to a further reduction in transport cost compared to standard control methods that would otherwise require additional time and energy to correct for increased navigation error caused by ocean current drift. In the event that exceptionally strong adverse or cross-track currents are encountered, the glider may not be capable of maintaining the intended path, and contingency planning may be initiated by the higher-level mission planning system.

To perform AVC, ocean current velocities must be known, and as a result, the DVL and science computer must be used to actively estimate these ocean currents. Thus, the AVC control policy is not serviceable for conventional low-power transit mode operation because conventional transit hotel load does not include DVL and science computer operation. This presents a trade-off that must be considered while transiting: utilize the conventional method of minimizing transit hotel load and forfeit the ability to perform AVC, or increase the hotel load during transit by operating the DVL and science computer to enable AVC. The value of this trade-off is dependent on environmental state. For example, if ocean current velocities have magnitude zero, it is unnecessary to operate the DVL and science computer because glider propulsion cannot be adjusted in response to ocean currents.

**Figure 6:**
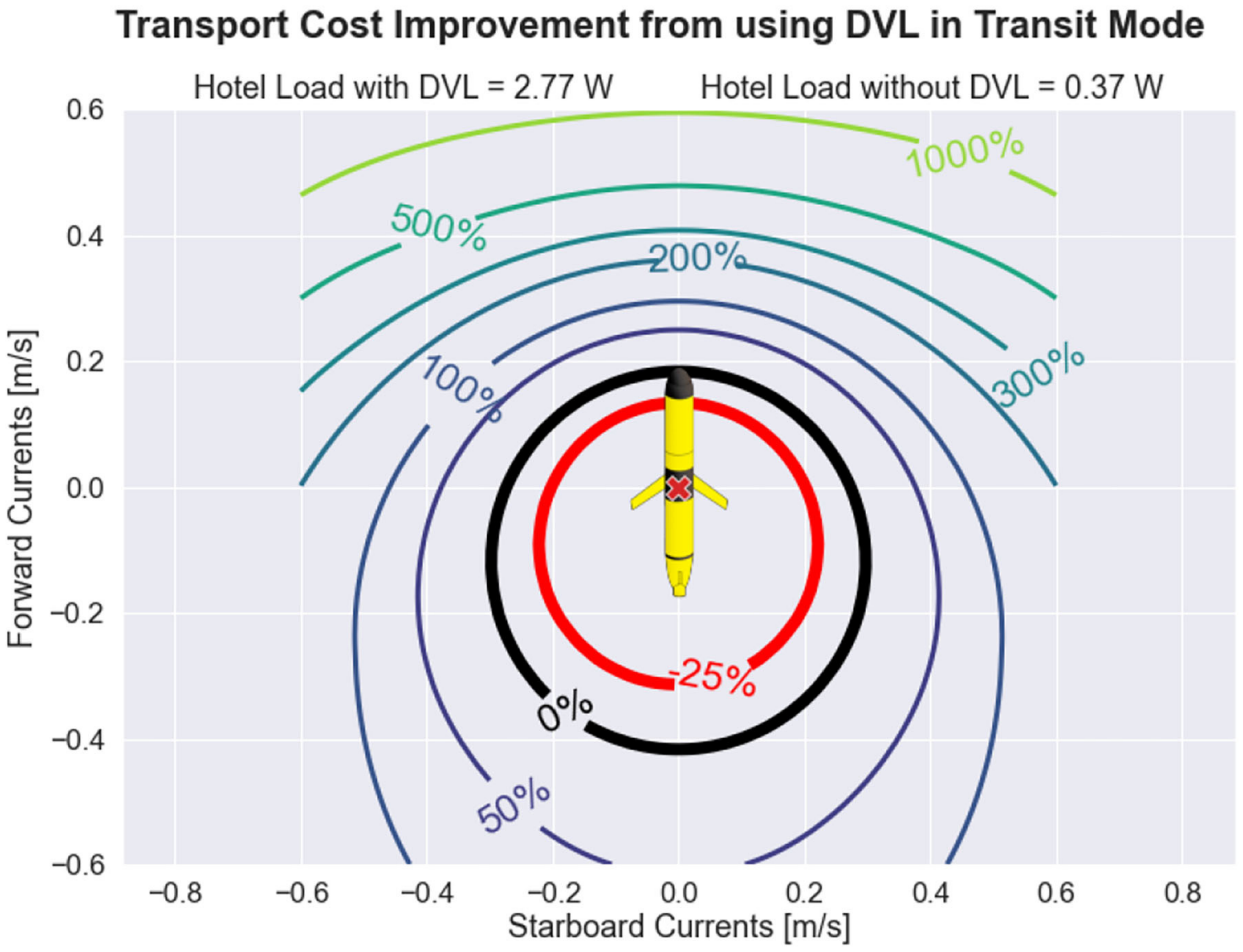
Transport cost improvement obtained by operating the Doppler velocity log (DVL) while transiting. By operating the DVL during transit, the autonomous underwater glider (AUG) can perform adaptive velocity control (AVC), which includes active steering to avoid cross-track and long-track drift caused by ocean currents and active throttling to take advantage of environmental conditions. The downside of operating the DVL is that the glider hotel load is increased. Thus, for ocean currents of small magnitudes, transport cost is actually worsened when the DVL is operated during transit, but for ocean currents of moderate magnitudes, transport cost can be substantially improved.

Figure 6 explores this trade-off by showing the percent decrease in transport cost when using the DVL and science computer while transiting, when compared to the default transit hotel configuration. The black solid line indicates when the glider transport cost is energetically equivalent between transiting with or without the DVL and science computer being operated. Inside the black solid line the transport cost associated with use of the DVL is increased and outside the black solid line the transport cost is decreased. Interestingly, the environmental state that leads to the greatest decrease in transport cost when using the DVL involves favorable ocean current with no cross-track component. This is largely due to the active steering component of AVC: the default transit mode overshoots the intended goal point due to long-track drift caused by favorable ocean currents, requiring a navigation correction that must then backtrack while fighting against ocean currents that are now entirely adverse to reach the goal point.

### 2.5. Exploitative Depth Band Selection

With sufficient available water depth, the glider can adaptively exploit velocity shear structure in the water column to minimize transport cost. This process is referred to as *exploitative depth band selection* (EDBS). As an example, if the water column contains two water mass layers with one layer having a favorable current and the other layer an adverse current, it may be energetically optimal for the glider to confine itself to the favorable layer instead of traveling throughout the entire available water column. The downside of this narrowing of depth band is an increased frequency of inflections, which corresponds with an increased usage of the energetically expensive buoyancy pump. This trade-off presents an optimization to further improve the transport cost of the glider if water column velocity estimates are available.

To optimize the depth band of the glider, we first express the aggregate transport cost incurred by the glider throughout an inflection cycle as the total energy expended during the cycle divided by the total horizontal distance covered during the cycle. In this fashion, the aggregate transport cost becomes the time-integrated version of the instantaneous transport cost given by Equation (20). $P_{thr}^{*}\left(z\right)$ represents the energy-optimized thruster power consumption, and $∥{\text{V}}_{og,\psi }^{*}\left(z\right)∥$ represents the energy-optimized over-ground glider speed, both at depth *z*. Both of these optimized quantities are computed via application of Equation (20) using the corresponding ocean current conditions at depth *z*. The glider power-draw contributions, *P*_*buoy*_, $P_{thr}^{*}\left(z\right)$, and *P*_**h**_, along with the glider over-ground horizontal speed, $∥{\text{V}}_{og,\psi }^{*}\left(z\right)∥$, can then be integrated in time to arrive at the aggregate transport cost of the glider throughout an inflection cycle. Next, the variable of integration *dt* is replaced using the definition of over-ground vertical speed: $w_{og}=\frac{dz}{dt}$. The resulting bounds of the integral are then given by the dive-to depth *z*_*d*_ and the climb-to depth *z*_*c*_, which are the parameters that define the glider depth band. Finally, the energy-optimized glider depth band, DepthBand^*^, is given by the argument minimum of the resulting expression, as shown in the following equation:

(21) $$\begin{array}{l} {\text{DepthBand}}^{*}=\underset{z_{d},\;z_{c}}{arg min}\left[\frac{\frac{E_{pump}}{2}+\int_{z_{d}}^{z_{c}}\frac{P_{thr}^{*}\left(z\right)+P_{\text{h}}}{w_{og}\left(z\right)}\cdot dz}{\int_{z_{d}}^{z_{c}}\frac{∥{\text{V}}_{og,\psi }^{*}\left(z\right)∥}{w_{og}\left(z\right)}dz}\right] \end{array}$$

The expression in Equation (21) concisely captures an intriguing trade-off between the energy cost of using the buoyancy pump *E*_*pump*_ and the two water-column-integrated variables: power and over-ground speed. Note that the energy cost of the buoyancy pump is divided by two because the pump is only utilized during bottom inflections. To illustrate the expressivity of this result, exemplar scenarios are presented. If a vehicle has negligible buoyancy pump cost, *E*_*pump*_ ≈ 0, then the EDBS will simply select a depth band of infinitesimally small height that is centered about the most favorable current in the water column. A similar conclusion can be made for vehicles with exorbitantly high hotel loads: the water-column-integrated hotel load dominates the buoyancy pump cost regardless of the size of the depth band, so the optimal depth band centers on the most favorable current in the water column. On the other hand, if a vehicle has excessively high buoyancy pump energy cost, EDBS will maximize the depth band height to minimize the frequency of using the energetically expensive buoyancy pump. For the case of uniform water column currents in depth, EDBS again maximizes the size of the depth band because there is no shear layer to exploit. Finally, when there is high contrast between velocity shear layers in the water column, it may be optimal for the vehicle to restrict itself to a narrowed depth band within the most favorable shear layer. An animated application of EDBS is provided in the Supplementary Material section.

Since EDBS considers all valid combinations of dive-to and climb-to depths, the policy will always perform at least as well as the default behavior of utilizing the maximum available water column. Because EDBS can be run as a continuous optimization, this process does not require regional ocean model forcasts of ocean currents, and can instead utilize the vehicle's real-time DVL water column measurements to update its depth band as water column currents evolve. To improve the computational speed of EDBS, additional constraints can be used to bound the set of valid dive-to and climb-to combinations.

### 2.6. Ocean Current Sensing

To take advantage of AVC as an embedded process, it is necessary to estimate horizontal ocean current velocities onboard the glider in real time. Since onboard sensors record measurements in the relative vehicle frame, the glider must determine the relationship between the relative vehicle frame and the absolute world frame to estimate ocean currents in the world frame. The glider velocity in the relative vehicle frame is referred to as the through-water velocity **V**_*tw*_. The through-water velocity of the vehicle can be expressed as the difference between two velocity vectors in the absolute world frame: the velocity of the glider over-ground **V**_*og*_ and velocity of the ocean currents **V**_*oc*_.

If the glider is able to estimate both its through-water velocity and its over-ground velocity simultaneously, the ocean current velocity can be estimated by taking the difference of the two: **V**_*oc*_ = **V**_*og*_ − **V**_*tw*_. Due to the sawtooth nature of the glider trajectory, the through-water velocity can be measured via a combination of depth, pitch and compass sensors, as shown in Claus and Bachmayer (2015). This approach for measuring through-water velocity is particularly advantageous because depth, pitch, and compass sensors are relatively low power and accurate to better than 1 % of dynamic range, and angle of attack is well characterized for Slocum gliders (Jenkins et al., 2003; Cooney, 2011; Eichhorn et al., 2020). Alternatively, if a glider is equipped with a DVL, this can be used in conjunction with the previously mentioned sensors to directly estimate angle of attack.

The vertical component of the through-water velocity vector can be measured as a change in depth over a change in time.

$$\begin{array}{l} w_{tw}=\frac{\Delta z}{\Delta t} \end{array}$$

Then, using glide angle as a sum of the glider pitch ϕ and angle of attack α, ξ = ϕ + α, and heading measurement ψ, the through-water velocity in the horizontal plane can be written as:

$$\begin{array}{l} u_{tw}=\frac{w_{tw}}{\tan\xi }\sin\psi \\ \nu_{tw}=\frac{w_{tw}}{\tan\xi }\cos\psi \end{array}$$

Putting the three equations together, we have the through-water velocity estimate as a function of measurements from the pressure sensor, pitch sensor, and compass, which we refer to as the dead-reckoned (DR) estimate of through-water velocity.

(22) $$\begin{array}{l} {\text{V}}_{tw}={\left(\begin{array}{ccc} \frac{\frac{\Delta z}{\Delta t}}{\tan\xi }\sin\psi & \frac{\frac{\Delta z}{\Delta t}}{\tan\xi }\cos\psi & \frac{\Delta z}{\Delta t} \end{array}\right)}^{T} \end{array}$$

After obtaining an estimate for through-water velocity, the glider must determine the relationship between the relative vehicle frame and the absolute world frame to estimate ocean current velocities. The glider can do this via two primary mechanisms: using surface drift velocity obtained from successive GPS measurements during a brief time window prior to mission start or using bottom-track DVL velocities when the seafloor is within range of the DVL. For the surface drift case, **V**_*og*_ is measured by successive GPS fixes at known times when **V**_*tw*_ = 0 as the glider is floating in a Lagrangian manner, which leads to **V**_*oc*_ = **V**_*og*_. For the DVL bottom-lock velocities, the DVL instrument measures **V**_*og*_ by measuring the Doppler shift of acoustic ping returns from stationary seafloor surfaces. Then, ocean currents can be estimated by subtracting the through-water velocity: **V**_*oc*_ = **V**_*og*_ − **V**_*tw*_. Both mechanisms for establishing a reference to the world-frame are infrequently available during a glider mission, making direct observation of absolute velocity sparse. Therefore, successive accumulation of velocity shear is required to propagate absolute velocity estimates when a world-frame reference is not directly observable (Visbeck, 2002). Stated another way, this method of *velocity shear propagation* (VSP) enables estimation of vehicle velocity in the absolute frame at any point in the dive, even when bottom lock is unavailable.

$$\begin{array}{l} {\text{V}}_{DR}={\text{V}}_{og}-{\text{V}}_{oc}\left(z\right)\\ {\text{V}}_{DVL}={\text{V}}_{og}-{\text{V}}_{oc}\left(z+\Delta z\right)\\ {\text{V}}_{DR}-{\text{V}}_{DVL}=\Delta {\text{V}}_{oc}{|}_{z}^{z+\Delta z} \end{array}$$

VSP uses the difference between through-water velocity measurements to measure a velocity shear, where a velocity shear encodes the change in ocean current velocity across a specified change in depth. Specifically, a velocity shear is computed as the difference between the DR through-water velocity **V**_*DR*_ and the DVL water-track through-water velocity **V**_*DVL*_ at the same instance in time. This is possible because the DVL water-track velocity measurements are recorded at some distance Δ*z* away from the transducer head. By taking the difference of the through-water measurements at the same instance in time, the over-ground velocity terms cancel out, leaving behind the velocity shear term Δ**V**_*oc*_.

**Figure 7:**
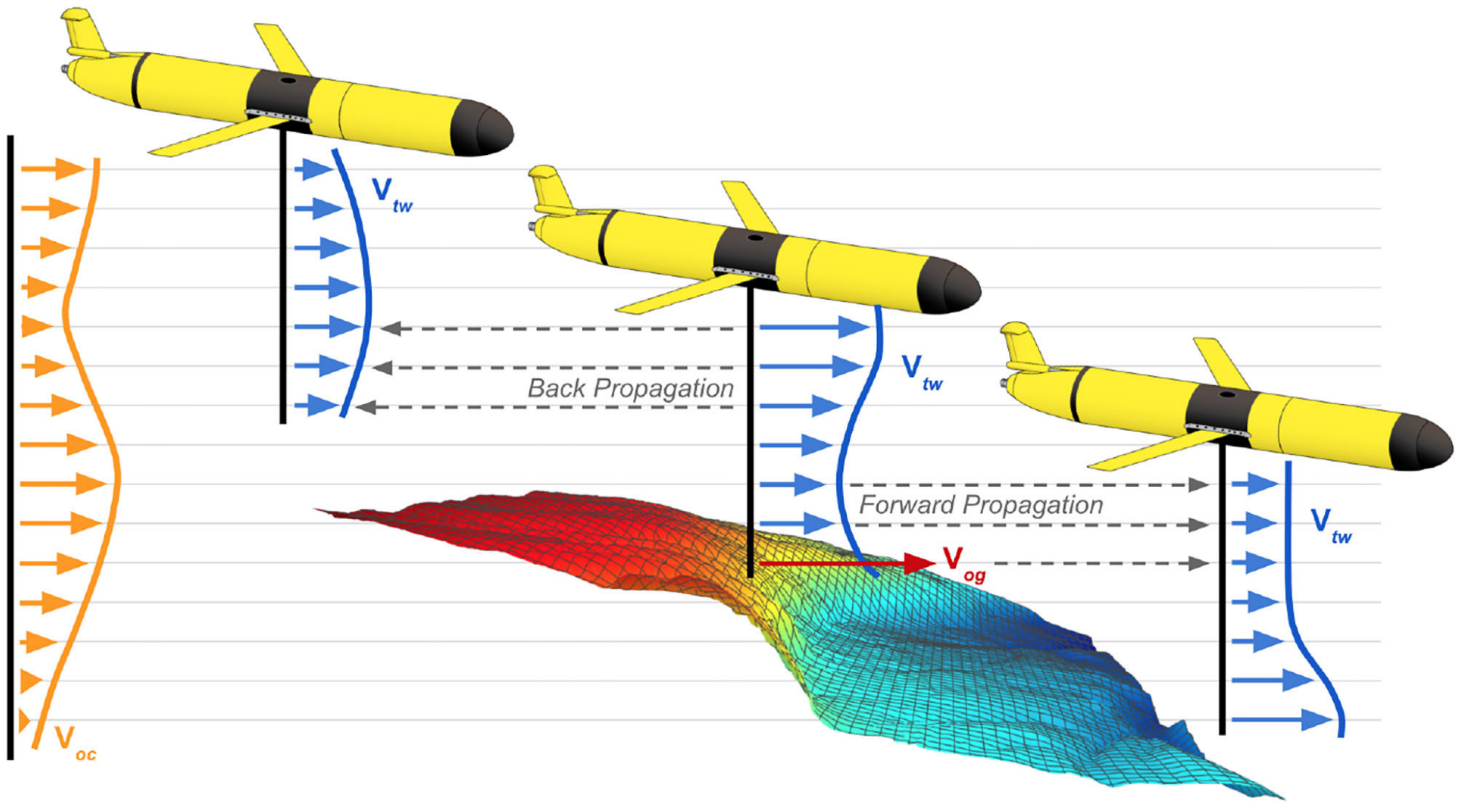
Schematic diagram of the velocity shear propagation (VSP) method being used to estimate water column currents. The VSP method uses forward propagation and back propagation to estimate ocean currents throughout the water column.

These velocity shear terms Δ**V**_*oc*_ can be incrementally added together via forward propagation to estimate water column currents in a relative frame. When an absolute velocity reference becomes available via GPS surface drift or DVL bottom-track, the absolute reference can be propagated (backward or forward) through the velocity shear measurements to estimate observed ocean currents and vehicle velocity in the absolute frame at any point in the dive, as shown in Figure 7. While this VSP method (Visbeck, 2002) and related least squares methods (Todd et al., 2017) have been used in post-process to provide more accurate estimates of water column currents for physical oceanography models, it can be applied in real-time to support Doppler odometry (Kinsey and Whitcomb, 2004). This real-time VSP capability extends DVL odometry beyond prior approaches, which required that bottom track be maintained (Woithe et al., 2011), freeing the glider to utilize the previously described AVC method even when operating at altitudes beyond the range of bottom track.

### 2.7. Sea-Ice Sensing

Measurements from an upward-looking region of the mechanical scanning imaging sonar (MSIS) are integrated with vehicle pose and processed to detect the presence of ice, its relative distribution, and thickness (Duguid, 2020), additional analysis of directionally dependent Doppler frequency shifts in the received signal can be used to estimate sea state (i.e., wave amplitude, frequency, and direction) (Burgess et al., 2020) during deployment. While these characterizations are useful for understanding Arctic climate and sea-ice processes, they are critical to mission completion and vehicle survival. Specifically, the glider can use these environmental state estimates to generate contingency plans for surfacing, but this must be carried out as an online process onboard the vehicle in order to provide actionable information for the glider's automated decision processes. This requirement impacts the overall energy budget via appropriate duty cycling of sensing and control systems and satellite communication during vehicle surfacing. The duty cycling aspect of the MSIS is presented in section 2.8. Efficiency of satellite communication can also be improved through onboard sensor processing because only a small subset of abstracted and compressed sea-ice classifications is required to be sent over the energetically expensive satellite link rather than the full raw data complement. In the event of vehicle loss, the subset of uploaded data prevents total loss of data.

**Figure 8:**
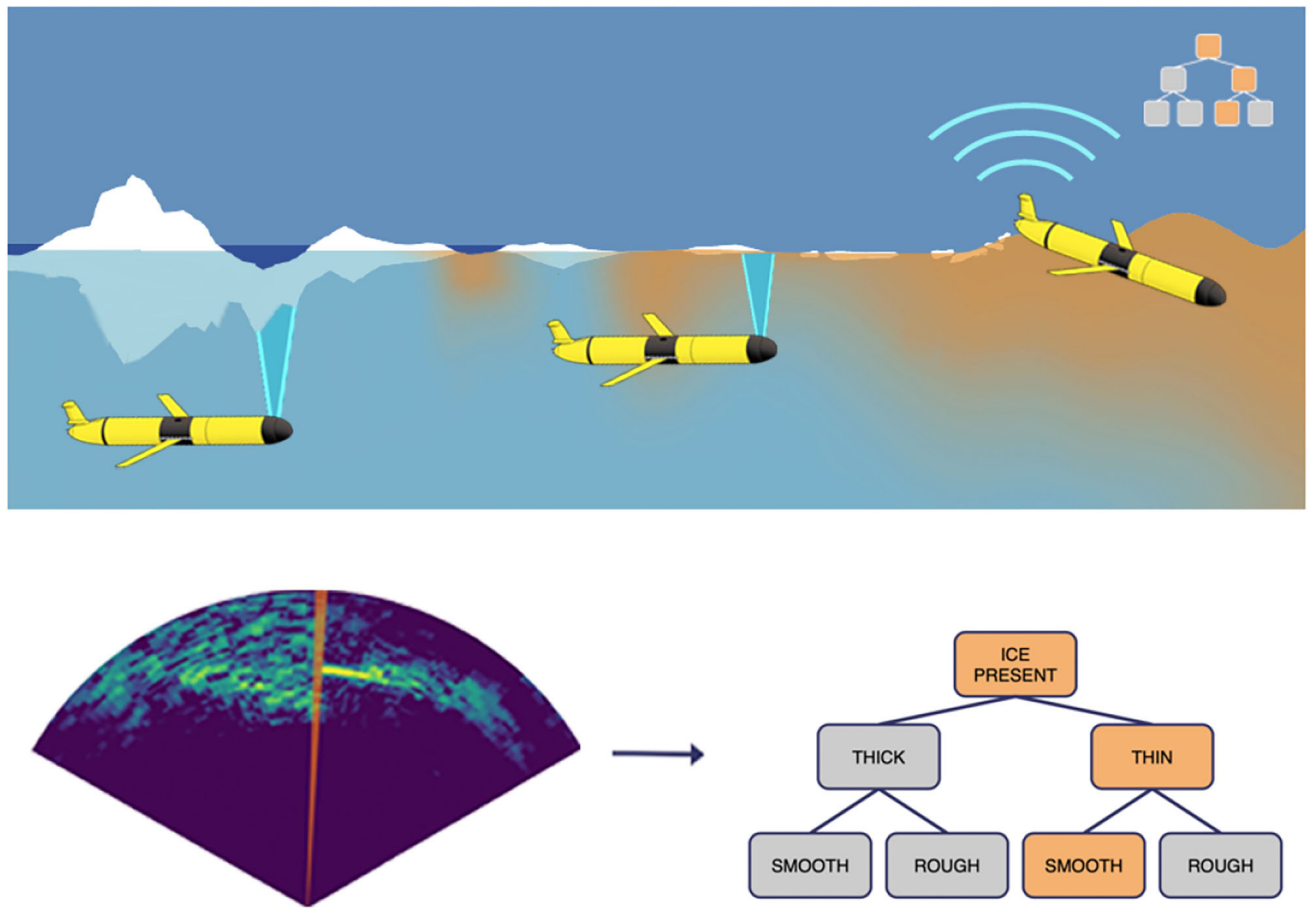
Schematic diagram of glider sampling sea-ice in the Arctic and transmitting sea-ice information via satellite communication. The glider processes a sequence of acoustic scan-lines in the form of an acoustic image, and then the onboard sensor processing module compresses the raw data into a hierarchical sea-ice characterization structure based on three primary feature: sea-ice presence, sea-ice thickness, and sea-ice roughness. The compressed classification is then transmitted to shore-side via periodic satellite communication during vehicle surfacing, which requires significantly less time and energy than communicating the raw sea-ice data complement. Figure adapted from Lee et al. (2012).

In this glider architecture, an MSIS collects data at a rate of approximately 8 kB per second and requires 15 s to complete a scan across the most informative ±60° sector for sea-ice classification, generating a sea-ice scan that is approximately 125 kB in size. In order to accommodate the decision input requirements of the glider's embedded contingency planner and the bandwidth limitations of the satellite communications, an automated sea-ice characterization process can perform a hierarchical analysis of sea-ice features, including the presence or absence of sea-ice, sea-ice thickness, and sea-ice roughness, as shown in Figure 8. From these three sea-ice features, sea-ice scan measurements can be categorized as a discrete set of sea-ice types (Duguid, 2020).

The satellite communication system requires 6.5 W of power when active. At the signal-to-noise levels typically available in the Arctic, the satellite communication system would be expected to transmit no more than 240 bits per second reliably. The automated sea-ice characterization process can, however, interpret and encode raw acoustic data from each scan into 8 bytes, which constitutes a four orders of magnitude size reduction, providing an equivalent decrease in time and power required to transmit sea-ice information via satellite communication. It is noteworthy that even if the glider had access to unlimited power, the data transfer rate for transmitting raw data would eclipse the collection rate by over 1.5 orders of magnitude, meaning that each minute of data collected would require over an hour of satellite-base transmittal. Thus, automated sea-ice interpretation and information compression is a necessity for sonar-based glider survey of Arctic sea-ice.

### 2.8. Adaptive Duty Cycling of Hardware

Although hotel load is often considered as a static differential between transit mode and survey mode, individual hardware components can be optimized with respect to vehicle and environment state in a process referred to as *adaptive duty cycling* (ADC). In this section, we consider the duty cycle reduction of three of the most power-intensive hotel components: the DVL, the MSIS, and the Back-seat Driver computer (BSD). Typically, these instruments are in continuous operation during glider missions. However, for resource-constrained glider missions such as those envisioned for the Arctic, operation of these instruments can be reduced to save power while still meeting safety and science data collection requirements. Additionally, we consider reduction of buoyancy engine duty cycle by decreasing the pumped volume of the buoyancy engine and by pursuing a shallower pitch angle of 5° to limit frequency of inflections.

The constraint that limits the minimum DVL duty cycle pertains to glider localization and ocean current velocity estimation. For both of these functions to be performed with reasonable accuracy, it is necessary for the DVL to make at least 10 water-track observations for each vertical meter traveled by the glider. At a nominal speed of 0.75 m s^−1^ at 5° pitch, the DVL must take samples at a frequency of 0.65 Hz. The nominal sampling frequency for the DVL at 100 % duty cycle is approximately 2 Hz. Therefore, to minimize DVL usage while meeting localization and state estimation requirements, the DVL can be operated at a duty cycle of 32 %, reducing its power draw from 2 W down to 0.64 W.

For duty cycle reduction of the MSIS, we enforce the science constraint that sea-ice classifications are binned at a spatial resolution of 50 m, with each bin composed of approximately 80 pings in order to provide a sufficient sample population for statistically reliable classification of sea surface regions because surfacing decision processes place an emphasis on minimizing false negative ice classification errors (Burgess et al., 2020; Duguid, 2020). From section 2.7, each sea-ice sonar scan is conducted at a depth of less than 50 m and takes 15 s to record. At a nominal speed of 0.75 m s^−1^ at 5° pitch, it takes the glider approximately 67 s to travel 50 m. Thus, to minimize the usage of the MSIS while adhering to the sea-ice sampling constraint, duty cycle of the MSIS can be set to 22 %, reducing the power draw from 3 W down to 0.66 W.

Finally, the BSD computer must be in operation to command the hotel load sensors such as the DVL and the MSIS, as well as process sensor data from these instruments in real-time and send glider control commands as necessary. We estimate that the BSD computer can be operated at 50 % duty cycle while maintaining proper coordination of glider sensors, data processing, and glider control, thereby reducing the power draw from 0.4 W down to 0.2 W.

This ADC strategy reduces cumulative power draw for the hotel load from 5.4 to 1.5 W. These duty cycle modifications could, in principle, be set as static parameters prior to the start of a mission, or adjusted dynamically in response to vehicle and environmental state. Further reduction in energy consumption can be realized when using the hybrid thruster by decreasing the pumped displacement volume of the buoyancy engine. For example, if the buoyancy engine, which requires approximately 2.95 W h per bottom inflection for full volume displacement, instead pumps just 20 % of its available volume, the duty cycle would decrease by 80 % resulting in 0.59 W h per bottom inflection. Despite glide efficiency decreasing with decreased pitch angle, additional duty cycle minimization may be obtained by decreasing the vehicle pitch angle to shallower angles around 5° that remain above the critical stall angle. While the exact percentages of duty cycle reduction are dependent on specific operating constraints of hardware components, significant energy reduction can be achieved through adaptive minimization of buoyancy engine and hotel system power draw without sacrificing science or mission requirements. As illustrated in section 4, a duty cycle reduction of this magnitude can lead to substantial improvements to glider range and mission duration.

## 3. Results

Our methods for propulsive control with AVC and ocean current estimation with VSP are evaluated using glider sea-trial data from a November 2019 deployment within an active submarine volcano in the southern Aegean Sea, which provided a complex testing environment with highly variable bathymetry and currents, as well as numerous hazardous obstacles. During these missions, the glider relied on its default DR process, which included a static current correction that calculated a temporal and depth-averaged water column current based on discrepancy between the GPS and DR localization estimates during its prior dive. This DR with depth-averaged current correction (DR-DACC) does not directly observe ocean current velocities and is unable to account for temporally dynamic currents (Eichhorn et al., 2014) or biasing caused by variability in the glider's depth band. Six glider missions are selected to evaluate the AVC method, and a subset of these missions are used to illustrate the performance of the VSP ocean current estimation method when bottom track is sparse or entirely absent. The methods described in this paper were not running in real time onboard the glider during these missions, but rather are evaluated in post-process.

### 3.1. Environment Sensing

**Figure 9:**
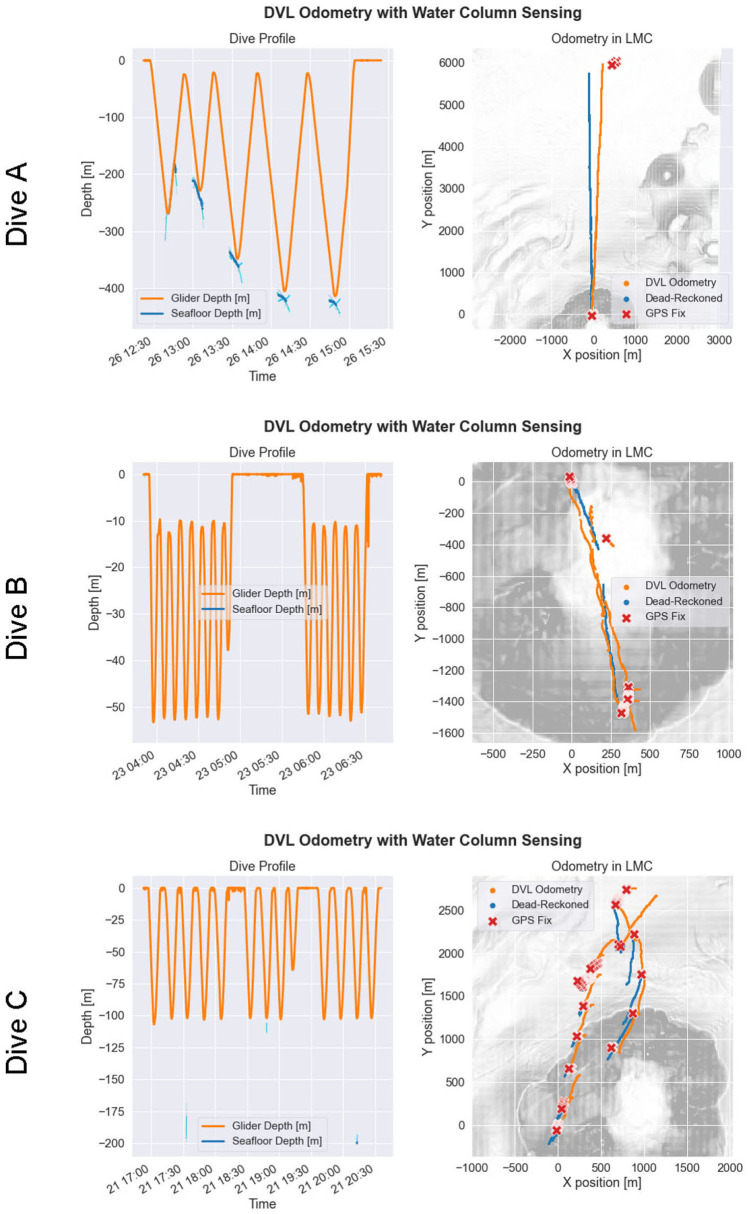
Post-process navigation reconstruction for a selection of glider missions where Doppler velocity log (DVL) bottom track was sparse or absent. Each row of the figure corresponds to a particular glider mission. The first column shows the glider depth (orange) and estimated seafloor depths (shades of blue) over time using each of the four DVL beams. The second column shows the dead-reckoned odometry from the glider flight computer (blue), the improved DVL-reinforced odometry (orange), and the GPS position updates (red), where the glider starts each mission at the origin of the local mean coordinates (LMC) coordinate system. The gray-scale underlay shows bathymetry.

**Figure 10:**
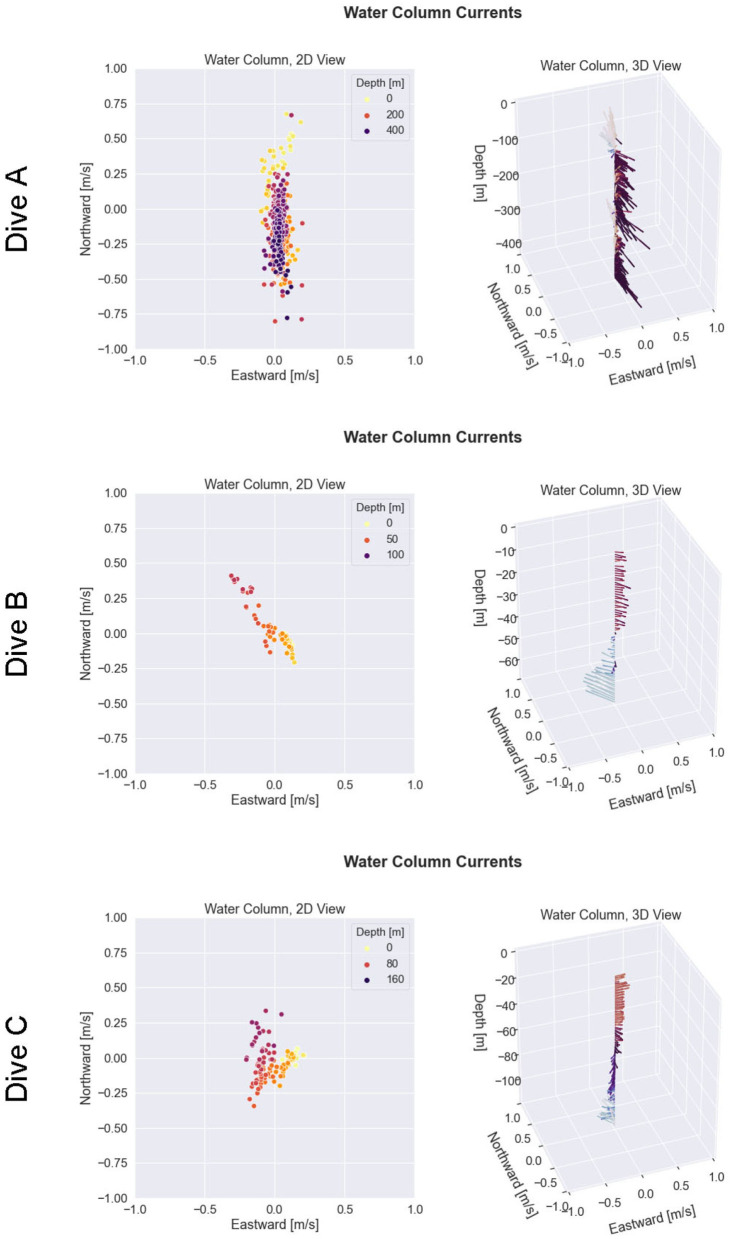
Post-process reconstruction of water column currents for the glider missions shown in Figure 9. Each row of the figure corresponds to a particular glider mission. The first column shows the observed water column currents in a 2D view. The second column shows the observed water column currents in a synoptic 3D view.

Results of the ocean current estimation process are shown in Figures 9, 10 for three example cases of dive missions with little-to-no bottom track availability due to the glider's altitude exceeding the DVL's maximum range for bottom track. Accuracy of DVL-reinforced odometry (DVL-Odo) is confirmed by comparing the odometry estimates to GPS position updates when the glider arrived at the surface. It is important to note that DVL-Odo is not performing Terrain-Aided Navigation (TAN) with known bathymetry maps. Instead, DVL-Odo estimates the water column currents with the VSP method according to section 2.6, and these water column currents are used to improve estimates of over-ground velocity **V**_*og*_. Although we do not have access to independent water column currents observations to compare against as ground truth, the performance of the DVL-Odo relative to GPS ground-truth provides corroborating evidence that the water column current estimates are valid.

In Dive A, the glider traveled due north for approximately 6,000 m, while staying submerged underwater for approximately 2.5 h. After surfacing, the DVL-Odo indicated a localization error equal to 5.6 % of distance traveled, whereas DR-DACC indicated a localization error of 11.9 % of distance traveled. The glider observed a strong southward ocean current, approaching 0.5 m s^−1^ in magnitude, for the majority of the water column, along with a small northward countercurrent near the sea surface. In Dive B, the glider identified a south-eastward current in the upper 40 m of the water column and a north-westward current between 40 and 80 m. Dive B included 2 GPS surface updates during the mission, and DVL-Odo indicated a mean localization error of 19.2 % of distance traveled while DR-DACC indicated a mean localization error of 54.3 % of distance traveled. In Dive C, the glider identified a low magnitude westward current throughout the water column, as well as a higher magnitude westward current at approximately 100 m depth. Dive C included 10 GPS surface updates during the mission and DVL-Odo indicated a mean localization error of 19.6 % of distance traveled, while DR-DACC indicated a mean localization error of 67.1 % of distance traveled.

In summary, when compared to DR-DACC, the DVL-Odo method typically decreased localization error by more than a factor of three. Had the glider not been using DR-DACC, the DR localization error would have exceeded 100 % of distance traveled for most dives. Furthermore, DVL-Odo is extrinsic to the specific DR process, enabling it to function regardless of if a depth-averaged current correction or similar correction was made, and does not require access to external acoustic navigation aides or uninterrupted observation of DVL bottom-track. Thus, although a detailed analysis of the glider localization process is beyond the scope of this paper, the glider missions highlighted in Figures 9, 10 demonstrate that DVL-Odo is able to reliably derive accurate position updates while submerged, without reliance on external navigation aids. The performance of DVL-Odo supports the validity of the VSP water column current estimation method and these real-time water column estimates can serve as inputs for AVC method described in section 2.4.

### 3.2. Adaptive Velocity Control

Based on the demonstrated ocean current estimation process, the potential utility of AVC can now be explored. Although the AVC method was not running onboard the glider, potential performance benefits of using this control method can be evaluated through offline analysis of three glider control policies: constant velocity buoyancy drive, constant velocity hybrid drive, and adaptive velocity hybrid drive.

**Figure 11:**
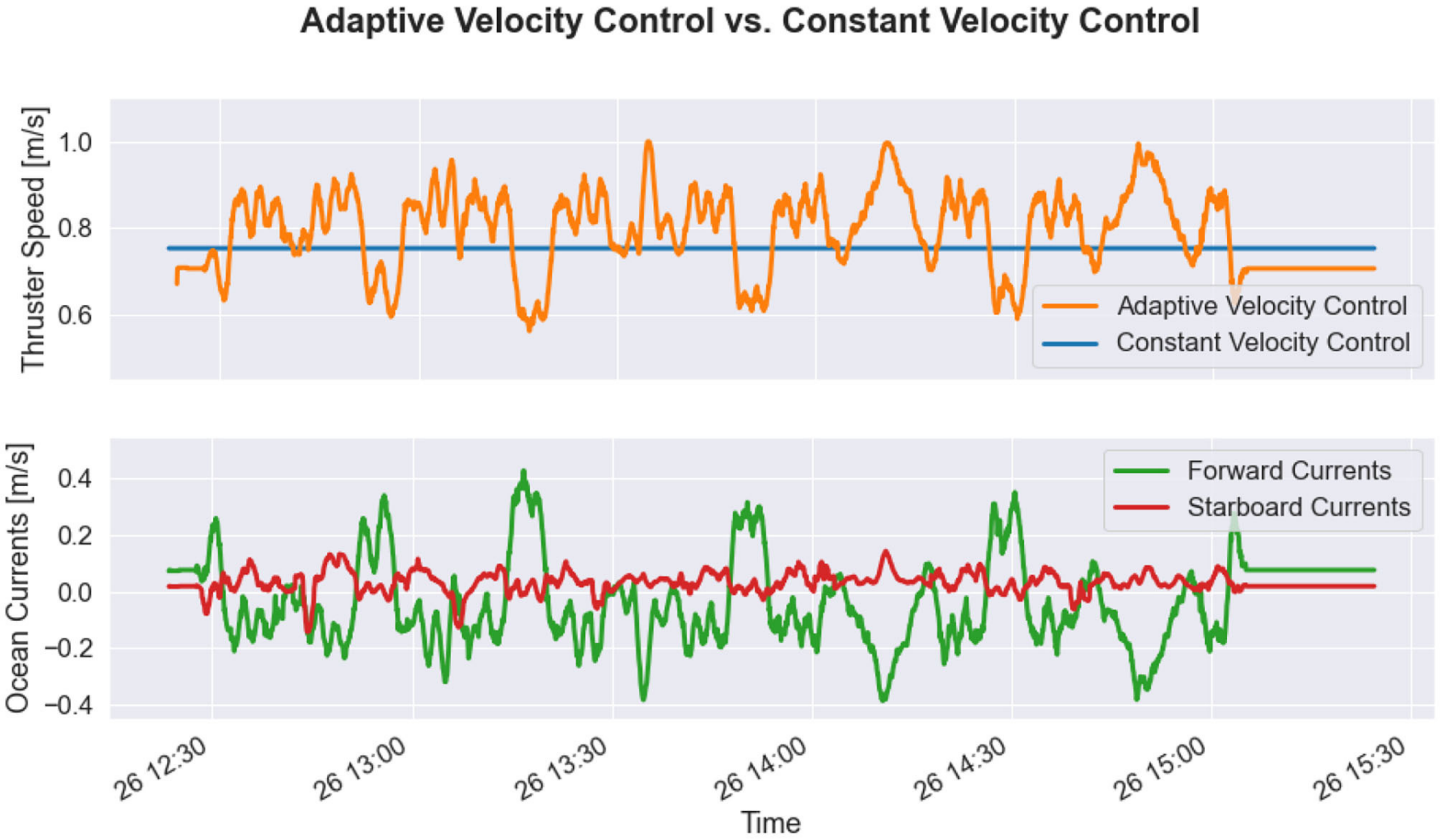
Adaptive velocity control (AVC) when applied to Dive A of the November 2019 deployment in the southern Aegean Sea. The hybrid adaptive speed curve (orange) shows the optimal glider speed over time, which responds to varying ocean current conditions. The hybrid constant speed (blue) is only optimized with respect to hotel load, so it does not change during the mission. The forward currents (green) and starboard currents (red) during mission are estimated via the velocity shear propagation (VSP) method. In periods of strong favorable currents, the glider slows down, while in periods of strong adverse currents, the glider speeds up, thus adaptively taking advantage of environmental state.

Figure 11 shows the behavior of the AVC method when applied to the Dive A series previously described in section 3.1. As shown in this figure, the glider increases its propulsive speed in times of adverse or cross-track currents, whereas the glider decreases propulsive speed in times of favorable currents. Since the majority of Dive A consisted of adverse ocean currents, the AVC would characteristically command the vehicle to a faster through-water speed than the constant velocity controller. While traveling at higher speeds increases the average power consumption of the glider, the mission duration is decreased such that the total energy consumption is reduced.

Table 2 shows the results of the three velocity control policies when applied to six glider missions conducted in November 2019 in the southern Aegean Sea. The velocity control policies are evaluated with respect to three metrics: mission duration, average power draw, and transport economy. Since the transport economy metric normalizes for the distance traveled during the mission, it is the most informative metric when comparing across the different missions.

**Table 2:** Offline performance analysis between three glider velocity control policies applied to November 2019 southern Aegean Sea missions previously mentioned: constant velocity with the buoyancy engine alone, constant velocity with the hybrid buoyancy and thruster system, and adaptive velocity control (AVC) with the hybrid buoyancy and thruster system.

**Mission name**	**Distance traveled [m]**	**Mission time [min]**		
		**Buoyancy constant speed**	**Hybrid constant speed**	**Hybrid AVC speed**
Dive A	6,100	319	160	**138**
Dive B	2,520	170	84	**83**
Dive C	4,910	261	132	**127**
Dive D	2,050	104	51	**48**
Dive E	905	62	24	**20**
Dive F	985	71	26	**22**
**Mission name**	**Distance traveled [m]**	**Average power draw [w]**		
		**Buoyancy constant speed**	**Hybrid constant speed**	**Hybrid AVC speed**
Dive A	6,100	**8.5**	12.2	13.4
Dive B	2,520	**18.3**	22.1	22.1
Dive C	4,910	**13.2**	17.0	17.3
Dive D	2,050	**13.2**	17.0	17.6
Dive E	905	**18.3**	22.1	23.3
Dive F	985	**18.3**	22.1	23.6
**Mission name**	**Distance traveled [m]**	**Transport cost [Jm**^**−1**^**]**		
		**Buoyancy constant speed**	**Hybrid constant speed**	**Hybrid AVC speed**
Dive A	6,100	25.6	18.7	17.6
Dive B	2,520	49.0	29.1	28.9
Dive C	4,910	35.7	23.1	22.7
Dive D	2,050	38.4	24.5	23.6
Dive E	905	75.9	35.3	32.2
Dive F	985	80.6	36.4	32.7
Mean transport cost		50.9 ± 20.6	27.8 ± 6.4	26.3 ± 5.5

*The control methods are evaluated with respect to three metrics: mission time, average power draw, and transport cost. The most important metric for glider energy efficiency, transport cost, shows that in this mission context the hybrid glider performs approximately twice as well compared to the buoyancy engine alone, and the AVC method reliably outperforms the constant velocity controller. Values in bold indicate best performance*.

As seen in the table, the hybrid glider achieves a transport cost that is approximately twice as efficient as conventional buoyancy drive. Although the buoyancy engine alone yields the lowest average power draw, the slower speeds of the buoyancy engine travel lead to significantly longer mission times, which leads to less overall energy efficiency.

Additionally, because the measured water column currents were consistently non-zero, the adaptive hybrid controller reliably outperforms the constant hybrid controller, regardless of the magnitude and direction of water column currents. This result confirms that transport cost can be independently optimized as a decoupled component of the glider mission planning problem. The adaptive hybrid controller performs 4.6 % more efficiently than the constant hybrid controller, and in instances of strong adverse ocean currents for large portions of the mission, such as in Dive A, the adaptive speed controller performs as much as 8.2 % better.

Therefore, by using Doppler acoustic sensing to estimate ocean currents in real-time onboard the glider, the vehicle can perform AVC to reap significant improvements in energy efficiency. In addition to AVC, further energy management improvements can be realized by modulating total displaced volume pumped by the buoyancy engine in relation to thruster power, and depth band can be continuously adjusted to maximally exploit favorable shear layers in the water column.

## 4. Discussion

Although the Slocum mission concept originally proposed by Stommel for gliders over 30 years ago was focused on high-endurance physical oceanographic studies of the water column (Stommel, 1989), technological advancements in battery chemistry, hybrid thrust, and low-power computation and sensing can enable glider mission scenarios for high-endurance unattended missions in confined environments, such as sea-ice surveys in the Arctic. The dangers of Arctic under-ice missions emphasizes on-board sensor interpretation, adaptive operation, and careful resource management. From an historical perspective, these constraints are not new. The glider control policies that we propose build upon a continuum of technological advancement relying on opportunistic scavenging of low potential environmental energy, which has been understood at least since the first sail was affixed to a boat. Identification of ocean currents to improve velocity-made-good performance is clearly documented as far back as Benjamin Franklin's analysis of sailing routes around the Atlantic Gulf Stream (Poupard and Franklin, 1786). More recently, thermal gliders (Webb et al., 2001) and wave gliders (Hine et al., 2013) have demonstrated the ability to generate sufficient thrust exclusively from ocean thermal gradients and kinetic surface energy, respectively, to propel robotic vehicles across entire marine basins. While the resource management policies that we propose here are potentially useful in areas where other energy-scavenging platforms currently operate, they are more expansive in context. Specifically, these glider resource management policies can provide a method for efficiently scavenging environmental energy while under sea-ice cover, where there is no opportunity to utilize wind, thermal gradients, or surface wave energy. Thus, with only modest modification to a legacy glider design, it may be possible to greatly extend its range and observational capacity to enable low-cost, persistent, and unattended survey of marine polar regions.

**Figure 12:**
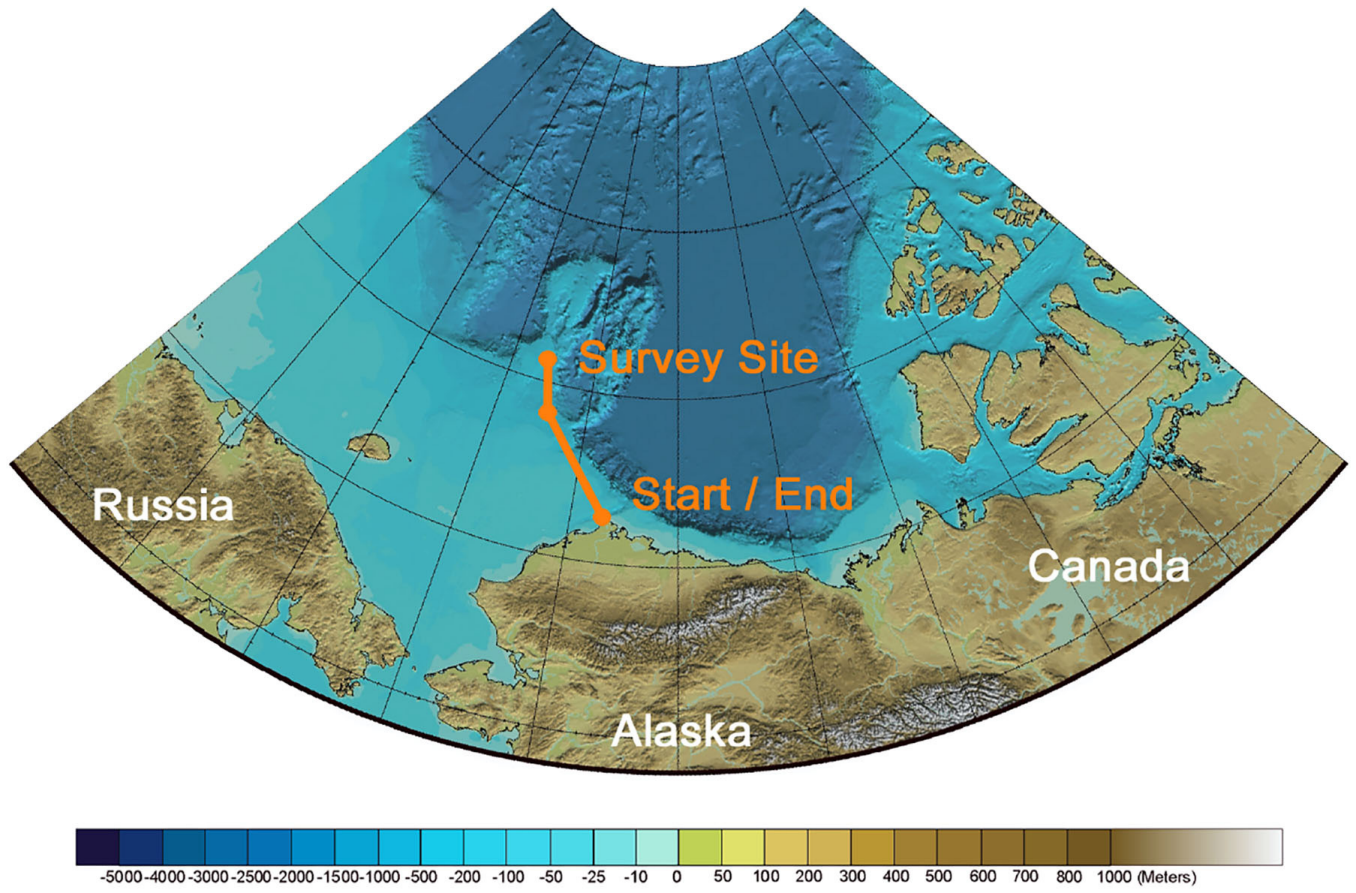
Overview of unattended glider sea-ice survey mission scenario during the spring-summer sea-ice retreat. For this mission, the glider departs Utqiaġvik, Alaska, transits 456km to the survey site on the Chukchi Plateau, performs a sea-ice survey, and then transits 456km back to Utqiaġvik. The feasibility of this mission depends on the resource management of the glider. Figure adapted from bathymetric map by Jakobsson et al. (2012).

With this scenario in mind, we can examine the possibility of a presently difficult-to-impossible science mission: persistent unattended observation of the MIZ and associated underlying water column temperature profile during seasonal sea-ice advance/retreat. During this time period, the MIZ may migrate more than 40 km d^−1^, outpacing the speed of conventional buoyancy-driven gliders, potentially trapping and crushing them. If, for example, the marginal ice zone survey area were located at the Chukchi Plateau [75.5°N, 164.0°W], and the glider start and end point is located near Utqiaġvik Alaska, which is the closest population center, the round-trip transit would be 913 km. In this scenario, as shown in Figure 12, the transit path along the continental shelf margin has a typical water column depth of approximately 100 m, enabling bottom lock DVL odometry, but the survey area includes regions with depths ranging beyond 1,000 m, necessitating use of a glider capable of deep operation. In this hypothetical mission scenario, let us assume that water column currents in the transit path have shear flow characteristics commonly found at continental margins, equivalent to the shear flow encountered during Dive B shown in Figure 10, and that water column currents in the survey region include an additional velocity component in the deeper region of the water column generated by the anticyclonic Beaufort Gyre at 315° azimuth with a 0.1 m s^−1^ horizontal velocity, as described by Plueddemann et al. (1998). Using these environmental state assumptions, the estimated mission duration and total range of scientific survey can be calculated for a conventional 1,000 m depth-rated buoyancy-driven glider and for an identically equipped hybrid glider utilizing various adaptive control policies.

Under the conditions of this mission scenario, we find that a conventional mission using a buoyancy-driven 1,000 m depth-rated Slocum glider operating with a 26° pitch angle would be unable to make the full round-trip transit, falling just short of a full round trip transit at 895 km, without having initiated an ice survey at the study site. In comparison, operating with a hybrid propulsion policy (buoyancy engine + thruster) operating at the hotel optimized velocity only improves range by order 100 km, but decreases the transit time by approximately 1 week. If the pitch angle is reduced to 12°, the buoyancy-driven glider would complete the round trip transit and a 310 km sea-ice survey, whereas hybrid thrust capability with this 12° pitch decreases transit time by a factor of 2 while increasing the total survey distance by over 200 km. However, implementation of the additional adaptive control policies, including the exploitative depth band selection (EDBS) and ADC during transit legs, enables the glider to complete the transit in approximately the same time as the 12° pitch hybrid constant speed policy, while extending the sea-ice survey by more than 2,700 km, as shown in Table 3. In summary, the use of hybrid propulsion that is optimized for hotel load in mission scenarios such as this (i.e., requiring shallow water transits in regions where variable currents are present) provides modest improvements in transport cost and transit time. Significantly greater improvements can, however, be realized through more advanced policies that optimize vehicle control and effectively harness environmental energy.

**Table 3:** Performance analysis of seven different glider control policies applied to a hypothetical sea-ice survey at the Chukchi Plateau.

**Glider control policy**				**Transit**	**Transit**	**Survey**	**Survey**	**Range Imp.**
**ID**	**Drive**	**Speed**	**Pitch**	****[*d*]****	**[Jm^**−1**^]**	****[*d*]****	****[*km*]****	**[%]**
1	Buoyancy	Constant	26°	28.6	40.1	—	—	—
2	Hybrid	Constant	26°	21.2	37.9	0.8	48	7.3
3	Buoyancy	Constant	12°	40.7	25.0	15.4	310	36.6
4	Hybrid	Constant	12°	19.9	23.0	8.5	515	59.5
5	Hybrid	AVC	12°	19.1	21.4	9.1	582	67.0
6	ADC	AVC	5°	22.4	6.1	61.4	3150	354
7	EDBS + ADC	AVC	5°	21.4	5.3	61.0	3260	366

*The survey mission includes 913 km round-trip transit, with remaining energy used for science data collection. The baseline glider control policy is a buoyancy-driven propulsion using 26° pitch; a buoyancy driven mission using 12° pitch is also examined. These propulsion policies are compared with a hybrid glider traveling at constant speed and a hybrid glider traveling at variable speed, via the *adaptive velocity control* policy, both of which follow a 12° pitch angle. Finally, we consider *adaptive velocity control* in combination with *adaptive duty cycling* and *exploitative depth band selection*, both of which follow a 5° pitch angle. Performance is evaluated with respect to transit time, transport cost, survey time and distance, and total range improvement compared with the baseline control policy of a buoyancy-driven glider at 26° pitch angle. Each successive control policy demonstrates continual improvement to performance*.

Although the EDBS is effectively a real-time 1D path planner that optimizes for bathymetric constraints and water column currents along the vertical *z*-axis, the low-level energy resource management processes described here can be combined with higher-level *xy*-plane path planners that incorporate water current forecast modeling, sensor scheduling, and risk assessment (e.g., Galea, 1999; Yu et al., 2013; Timmons et al., 2016). Prior path planning work based on satellite-observed surface water currents has demonstrated current-augmented vehicle speed over-ground in excess of 1 m s^−1^ during transoceanic crossings (Ramos et al., 2018), improving energy efficiency while minimizing travel time.

Looking toward the future of robotic under-ice survey, it is informative to consider that in addition to propulsion system inefficiencies, other glider inefficiencies stem from limitations in fabrication processes, which thereby constrain vehicle design. Although the resource management policies described here were applied to a legacy glider design, they may, in principle, be extended to other vehicle classes. As technological innovations in materials, manufacturing, and design advance, these policies should also be well-suited for next-generation designs that are more hydrodynamically efficient.

Under-ice survey of recently identified but distant ocean worlds of Europa and Enceladus is still the realm of science fiction, but may someday be within technological reach. For example, the NASA ICEE resource accommodation plan for a Europa surface lander limits the entire payload to just 32.7 kg, 1,600 W h, and 600 megabits for 20 days of stationary operations (Krajewski, 2018). Survey below the estimated 10 km thick Europan ice sheet (Billings and Kattenhorn, 2005) using a mobile platform will almost certainly require decreased payload accommodation, suggesting that propulsion/hotel load balancing will result in a platform with a mass and power budget less than, or at most equivalent to, prototype designs presently under consideration (Hildebrandt et al., 2013; Spears et al., 2016; Wirtz and Hildebrandt, 2016). Assuming a mission cost in excess of $100M at present currency values, this translates to a Europan survey platform costing more than $1M per kg. Incorporating onboard sensing and control policies that can opportunistically identify and scavenge kinetic environmental energy sources as described here, along with thermal/chemical gradients, could greatly reduce the required size of the power system, minimizing the vehicle system's mass while greatly extending mission life for under-ice exploration of these remote ocean worlds. Because the sensing and control policies described here are hardware independent, they are extensible to broad classes of vehicles, including conventional aerial platforms as well as systems currently under development, such as the *Dragonfly* lander (Lorenz et al., 2018), which is planned for an *in-situ* exploration mission of Saturn's icy moon Titan. Looking back toward polar Earth deployments, these innovations could provide an elegant approach for faster, more complete synoptic under-ice survey of polar marine environments without requiring scaling up in vehicle size, cost, and risk. As Jenkins et al. (2003) aptly recognize, failure to adapt vehicle behavior in response to environmental state “*can result in failure to exploit performance improvement offered up by Nature free of charge or can make even worse already catastrophic losses of performance*.”

## Data Availability Statement

The raw data supporting the conclusions of this article will be made available by the authors, without undue reservation.

## Author Contributions

ZD and RC developed models, analysis, and energy management policies presented here. ZD was responsible for energy management algorithm development. RC was responsible for *Polar Sentinel* glider design and operations. Field data presented in this paper rely on glider missions conducted during a November 2019 oceanographic research cruise with RC as chief scientist. ZD and RC authored this manuscript jointly.

## Conflict of Interest

The authors declare that the research was conducted in the absence of any commercial or financial relationships that could be construed as a potential conflict of interest.
